# Photocatalytic Aqueous Reforming of Methyl Formate

**DOI:** 10.1002/adma.202509890

**Published:** 2025-07-11

**Authors:** Dongxu Zuo, Suman Pradhan, Manami Banerjee, Nils Rockstroh, Stephan Bartling, Abdallah I.M. Rabee, Xinxin Tian, Alina Skorynina, Aleksander Jaworski, Laura Simonelli, Jabor Rabeah, Haijun Jiao, Matthias Beller, Shoubhik Das

**Affiliations:** ^1^ Department of Chemistry University of Bayreuth Universitatsstraße 30 95447 Bayreuth Germany; ^2^ Leibniz‐Institut für Katalyse e.V. (LIKAT Rostock) Albert‐Einstein‐Str. 29a 18059 Rostock Germany; ^3^ Chemistry Department Faculty of Science Minia University El‐Minia 61519 Egypt; ^4^ Institute of Molecular Science Key Laboratory of Chemical Biology and Molecular Engineering of Ministry of Education Shanxi University Taiyuan 030006 P. R. China; ^5^ CLAESS Beamline ALBA Synchrotron Light Facility Carrer de la Llum 2‐26, Barcelona Cerdanyola del Vallès 08290 Spain; ^6^ Department of Chemistry Stockholm University Stockholm 10691 Sweden; ^7^ State Key Laboratory of Low Carbon Catalysis and Carbon Dioxide Utilization Lanzhou Institute of Chemical Physics (LICP) Chinese Academy of Sciences Lanzhou 730000 P. R. China

**Keywords:** atomically dispersed photocatalyst, DFT calculations, green hydrogen, LOHC, photocatalysis

## Abstract

Green hydrogen is critical to establish a sustainable energy future as it offers a clean, renewable, and a versatile alternative for decarbonizing industries, transportation, and power generation. However, the limitations of current methods significantly restrict the scope and hinder many of the envisioned applications. This study aims to report on the first example of a 3d‐metal‐based (Cu) heterogeneous photocatalytic system to produce green hydrogen via dehydrogenation of methyl formate (MF), a reaction previously known to require 4d/5d transition metals. Employing a Cu‐based atomically dispersed heterogeneous photocatalyst supported on aryl‐amino‐substituted graphitic carbon nitride (d‐gC_3_N_4_), the protocol offers numerous key advantages, including the recyclability of the photocatalyst for >10 cycles without significant activity loss, sustained hydrogen production (>15 days!) with high hydrogen yield (19.8 mmol g_cat_^−1^) and negligible CO emission, following an operationally simple, sustainable, and efficient catalytic pathway. Furthermore, the photocatalyst is characterized (using HAADF‐STEM, SS‐NMR, XAS, EPR, and XPS), all of which clearly demonstrated the presence of single atomic Cu‐site. Additionally, comprehensive mechanistic investigations together with DFT calculations allow for a thorough mechanistic rationale for this reaction. It is strongly believed that this atomically dispersed heterogeneous photocatalytic approach will open new avenues for establishing liquid organic hydrogen career (LOHC) technologies.

## Graphical abstract

A 3d‐metal Cu‐based atomically dispersed photocatalyst, supported on modified g‐C₃N₄, facilitates the photocatalytic selective hydrogen (7.5 mmol g_cat_^−1^ of hydrogen; nothing else than H_2_ and CO_2_) generation from methyl formate. This diverse system functions under mild conditions, providing high hydrogen yield, selectivity, and enduring stability. Through characterization and mechanistic investigations uncover a radical approach, offering a sustainable foundation for the future advancement of liquid organic hydrogen carrier (LOHC) technologies utilizing earth‐abundant metals.

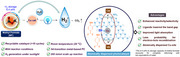

## 1 Introduction

Steam reforming is currently the most important industrial process for the production of “black” or “grey” hydrogen from fossil resources.^[^ ^1^, ^2^ ^]^ The process entails the reaction of hydrocarbons such as natural gas with steam at very high temperatures, typically in the presence of a catalyst. The process is characterized as endothermic, necessitating the application of significant heat to stimulate the reaction. The resultant mixture consists of hydrogen, carbon monoxide, and carbon dioxide. In order to achieve the desired level of purity in hydrogen, it is necessary to perform additional processing steps, including the water‐gas shift reaction and pressure‐swing adsorption techniques. In the context of a sustainable hydrogen economy in the future it is necessary to move away from these processes toward a carbon‐neutral hydrogen generation (“green” hydrogen).

**Figure 1:**
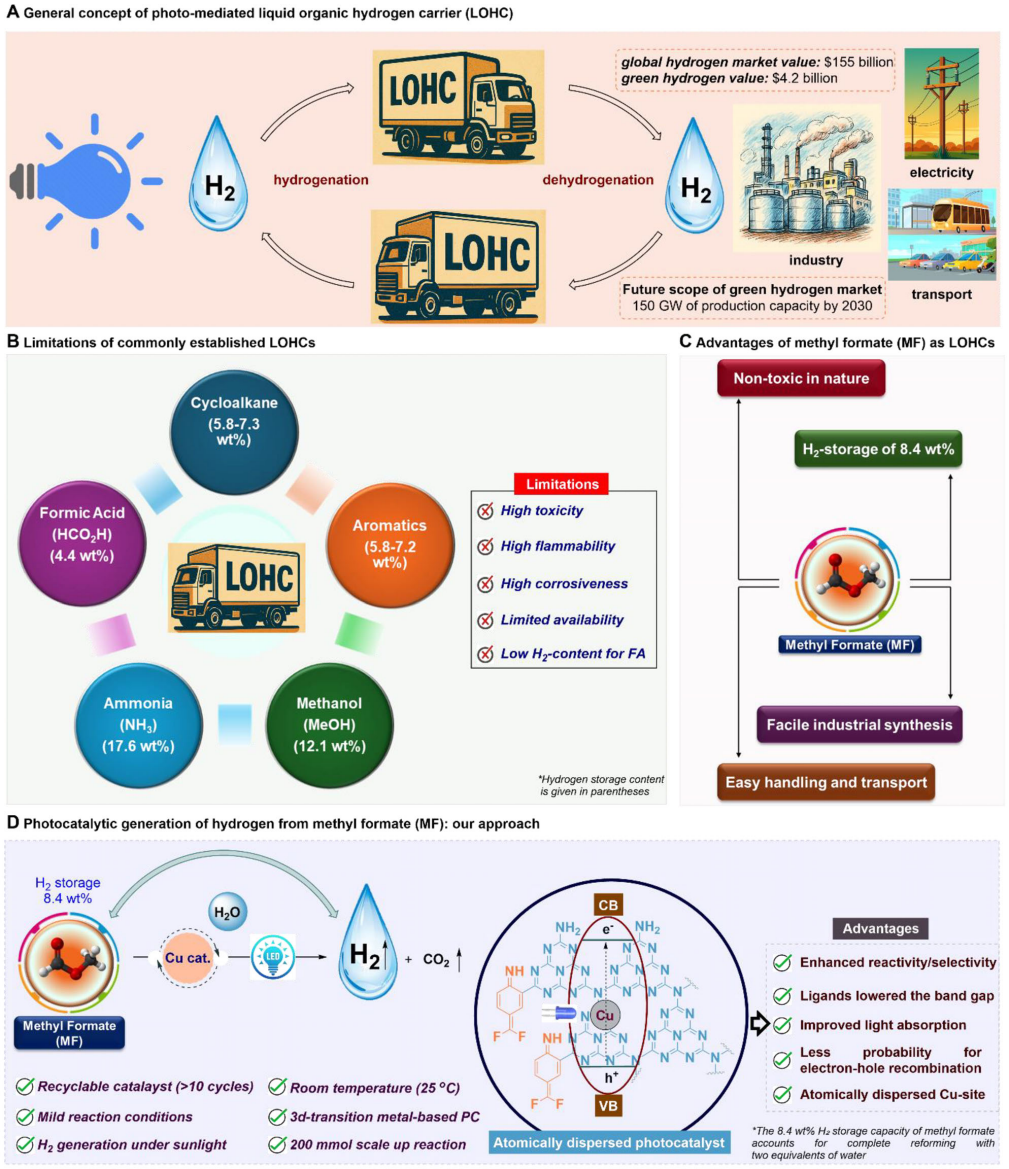
State‐of‐art for the generation of hydrogen from Liquid Organic Hydrogen Carriers (LOHCs). All images used here are exempted from copyright.

“Green” hydrogen is widely recognized as a crucial energy carrier^[^ ^3^, ^4^, ^5^ ^]^ in the transition toward a sustainable economy, with uses across multiple industries, including transportation, energy storage, and industrial operations.^[^ ^6^, ^7^ ^]^ However, due to unfavorable physical properties such as high flammability and low density under ambient conditions, storage and transportation have become challenging and that are prompting chemists to explore alternative carriers.^[^ ^8^, ^9^, ^10^ ^]^ In this scenario, Liquid Organic Hydrogen Carriers (LOHCs) (**Figure** **1**A) provide a safe and efficient method for storing and transporting green hydrogen, which is produced from renewable energy sources.^[^ ^11^, ^12^, ^13^ ^]^ In particular, LOHCs can be stored for extended periods of time than conventional fuels and can be easily transported in larger quantities over long distance via liquid fuel transportation strategies such as pipelines, ships, vessels and others.^[^ ^14^ ^]^

By integrating LOHC systems, the challenges of hydrogen storage and distribution are significantly reduced, facilitating a more sustainable hydrogen economy.^[^ ^13^, ^15^ ^]^ The current global hydrogen consumption is ≈97 million metric tons per year, and indicates that this figure will rise to ≈200 million metric tons by 2030 due to a very high demand for carbon‐neutral hydrogen.^[^ ^16^ ^]^ Therefore, at this stage, a rational selection of a LOHC, followed by an efficient generation of ‘green hydrogen’ from it, is urgently required to drastically reduce of the carbon footprint associated with hydrogen production.^[^ ^17^, ^18^ ^]^

An applicable LOHC must be non‐toxic compared to the common hydrocarbon‐based fuels, and should be easily available (from renewable resources) as well as must have high hydrogen storage capacity of >4.0 wt.%.^[^ ^11^, ^15^ ^]^ Additionally, selective hydrogenation and dehydrogenation processes must be ensured for the circularity and should undergo several cycles before their replacement.^[^ ^11^, ^19^ ^]^ Regarding potential candidates, methanol (12.1 wt.% H_2_ content),^[^ ^3^, ^20^ ^]^ and ammonia (17.6 wt.% H_2_ content)^[^ ^21^ ^]^ are commonly studied, but they pose severe challenges due to their toxicity and flammability.^[^ ^18^ ^]^ Furthermore, while formic acid (4.4 wt.% H_2_ content)^[^ ^8^, ^22^ ^]^ can be derived from CO_2_ and is easily dehydrogenated, its low hydrogen content and corrosiveness limits its practicality. On the other hand, cycloalkanes^[^ ^23^ ^]^ and *N*‐heterocyclic compounds have moderate hydrogen capacities of 5.8–7.3 wt.% and excellent handling properties, however, they are limited to larger application due to limited availability as well as toxicity (Figure 1B).^[^ ^14^ ^]^ This has led to increased interest in developing a new and non‐toxic hydrogen vector such as methyl formate (MF) which has a hydrogen storage capacity of 8.4 wt.%, making it a viable alternative with easier handling and transport capabilities.^[^ ^18^, ^24^ ^]^ Furthermore, the industrial synthesis of MF via carbonylation of methanol (global market size of MF was roughly 810 thousand tonnes in 2022 and is expected to reach 1200 thousand tonnes by 2032) enhances its attractiveness to use it as a hydrogen carrier.^[^ ^25^, ^26^ ^]^ Considering these advantages (Figure 1C), some of us have developed an excellent Ru‐based homogeneous catalyst which exhibited the generation of hydrogen from MF with a TOF of >8,300 h^−1^ at elevated temperature (90 °C).^[^ ^18^ ^]^ In the context of LOHCs, photocatalysis presents a highly attractive strategy for hydrogen release due to its potential for low‐temperature operation, which minimizes thermal degradation of the carrier molecule.^[^ ^27^ ^]^ Traditional dehydrogenation processes often require high heat, which can compromise the longevity and recyclability of the LOHC system. Photocatalytic methods, by contrast, offer a pathway to more energy‐efficient and mild dehydrogenation, thereby enhancing the overall sustainability and practicality of LOHC‐based hydrogen storage technologies.^[^ ^27^ ^]^ From a practical perspective, development of a heterogeneous catalyst should be advantageous in this domain since the heterogeneous catalyst will allow several cycles of dehydrogenation in parallel. Additionally, if this heterogeneous catalyst brings high reactivity as well as selectivity at room temperature by using light as a traceless reagent, that will be a significant enhancement in this blueprint. For this purpose, design of an atomically dispersed photocatalyst will be highly effective due to its inherent tendency to impart unique reactivity, recyclability, selectivity and an efficient atom utilization.^[^ ^28^ ^]^ However, the support materials, akin to ligands in organometallic complexes, play a crucial role in facilitating charge transfer and enhancing photocatalytic reactivity.^[^ ^29^ ^]^ In this respect, polymeric carbon nitrides (PCN) have emerged as an invaluable support material for designing robust atomically dispersed photocatalysts which offer exceptional stability and tunable chemical properties to enhance catalytic reactivity as well as precise control over reactivity.^[^ ^28^, ^30^, ^31^, ^32^ ^]^

Relying on these, we developed an atomically dispersed copper catalyst supported on functionalized graphitic carbon nitride (Cu@d‐gC_3_N_4_), leveraging its well‐established photocatalytic properties. This heterogeneous framework aims to enable the efficient and selective dehydrogenation of methyl formate to produce hydrogen and carbon dioxide (H_2_ + CO_2_) at ambient temperature, while completely suppressing the competitive formation of carbon monoxide and water (CO + H_2_O) as demonstrated in Figure 1D. Particularly, we have functionalized the parent g‐C_3_N_4_ core by the inclusion of an aryl amino moiety to lower down the bandgap for improving the light‐absorption and doping of low‐toxic, earth abundant Cu‐metal to inhibit the electron‐hole charge recombination. We argued that an atomically dispersed photocatalyst should effectively perform redox reactions by generating a hydrogen atom transfer (HAT) reagent such as hydroxyl radical (•OH) which can be utilized for the selective dissociation of a C─H bond in MF (BDE ≈ 97–102 kcal mol^−1^).^[^ ^33^, ^34^ ^]^ It is anticipated that •OH will be capable of abstracting a hydrogen atom due to their high bond dissociation energy (BDE ≈ 105 kcal mol^−1^ for O─H bonds),^[^ ^35^ ^]^ therefore, selective generation of •OH from OH⁻ (should be an alkaline solution such as KOH and NaOH) should ideally facilitate the C─H bond cleavage process.^[^ ^36^ ^]^ In general, the valence band (VB) of PCN‐based atomically dispersed photocatalysts lays in the range from +1.50 to +1.90 V (vs NHE)^[^ ^37^ ^]^ which should be sufficient to oxidize OH⁻ to generate •OH (*E_•OH/OH_^−^* = + 1.99 V vs NHE).^[^ ^38^ ^]^ We further rationalized that after the hydrogen atom abstraction from MF, an acyl radical intermediate (•COOCH_3_) will be formed which will recombine with •OH to produce HOCOOCH_3_. This species will then undergo deprotonation, further oxidation at the VB, and decarboxylation to form formic acid (FA). The deprotonation of FA followed by a single‐electron oxidation of HCOO⁻ at the VB should generate the HCOO• intermediate, that upon facile decarboxylation will release CO₂ and produce H‐atom (H•). The H‐atoms will undergo radical‐radical coupling, while protons (H⁺) from formic acid oxidation will be reduced at the conduction band (CB) (*E_CB_* of Cu@d‐gC_3_N_4_ was at ca. –0.9 V vs NHE) to produce H_2_.

## 2 Results and Discussion

### 2.1 Optimization of the MF Dehydrogenation Reaction

**Figure 2:**
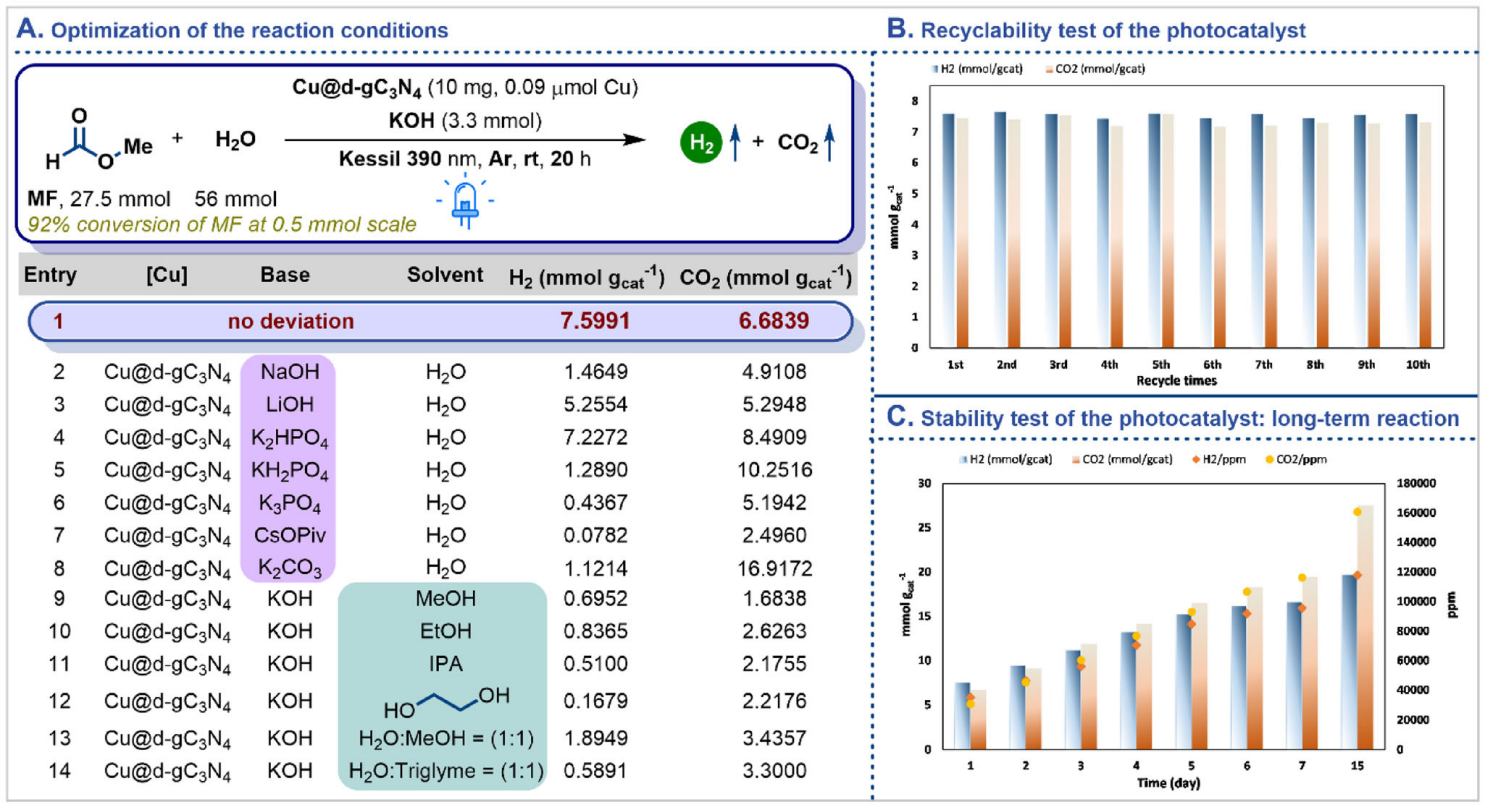
A) Optimization table; B) recyclability of the photocatalyst; C) long‐term reaction.

At the outset of this project, a mixture of MF, water, base, and a photocatalyst were irradiated using a Kessil lamp (λ = 390 nm) for 20 h in a 25 mL Schlenk tube at room temperature. After thorough investigations, it was found that the mixture of MF (27.5 mmol, 8.3 equiv.), water (56 mmol, 17 equiv.), KOH (3.3 mmol, 1 equiv.) and Cu@d‐gC_3_N_4_ (10 mg, with a Cu loading of 0.2 wt%, w.r.t. metal precursor which corresponds to 0.09 µmol of Cu) exhibited a remarkable performance (**Figure** **2**a, entry 1), producing 7.5991 mmol g_cat_^−1^ of H_2_ and 6.6839 mmol g_cat_^−1^ of CO_2_ (‘g’ refers to the amount of photocatalyst used) with a negligible amount of CO (78 ppm, 0.01638 mmol g_cat_^−1^). In fact, using small scale reaction i.e., 0.5 mmol of methyl formate, the system achieved a 92% conversion w.r.t. MF (includes all detectable products derived from MF) with the generation of H_2_ in similar amount (Table S10, Supporting Information). Various inorganic bases (Figure 2a, entries 2–8) were also investigated to improve the reactivity under these reaction conditions, however, no further improvement was observed, underscoring the critical roles of OH⁻ and K⁺ in the reaction. Although K_2_HPO_4_ demonstrated comparable H₂ production, KOH was selected as the optimal base for this system due to its lower CO₂ generation. Furthermore, the influence of basicity was evident in the reaction, with KOH exhibiting higher alkalinity yielding superior results compared to other bases. In parallel, substituting H_2_O by alternative solvents such as methanol, ethanol and others (Figure 2a, entries 9–14) also failed, highlighting the essential role of H_2_O (solubilizing the reaction components as well as promoting the reaction equilibrium for steam reformation of MF) as a solvent in this transformation (Figure 2a, entries 8–11). Notably, the production of CO_2_ consistently exceeded that of H_2_ in case of entries 5–14 due to the rapid decarboxylation of formic acid (which is generated as an intermediate) under the irradiation of visible light.^[^ ^39^ ^]^ Keeping the practical applicability in mind, the model reaction was conducted under the irradiation of direct sunlight as well as was scaled up to 200 mmol of MF under the irradiation of Kessil lamp. Both resulted in the production of H_2_ albeit with a reduced yield of 1.1186 mmol g_cat_^−1^ and 0.9845 mmol g_cat_^−1^ respectively (for details, see Tables S12 and S13, Supporting Information). Simultaneously, after optimizing the reaction conditions, the photocatalyst was recycled for >10 consecutive cycles, and demonstrated no significant loss of reactivity in hydrogen generation from MF (for detailed procedures, please refer to the Figure S13, Supporting Information). This excellent stability highlights the strong potential for applications of the photocatalytic MF dehydrogenation technique from an economic perspective. Subsequently, the stability of the photocatalyst was assessed by conducting a long‐term reaction of >15 days without isolating the photocatalyst from the reaction during this period. Gratifyingly, a simultaneous increase in hydrogen generation was observed, suggesting that the photocatalyst remained stable for >15 days.

### 2.2 Characterization of the Photocatalyst

After synthesizing Cu@d‐gC_3_N_4_, several characterization techniques were employed to examine its structural and electronic properties. At first, ultraviolet‐visible diffuse reflectance spectra (UV–vis DRS) and Mott‐Schottky measurements were collected to determine the electronic band structure of the photocatalyst (Figure S8, Supporting Information). The bandgap of Cu@d‐gC_3_N_4_ was determined to be 2.39 eV from the Tauc plot. The positive slope of the Mott‐Schottky curve suggested that the photocatalyst was *n*‐type. The flat band potential (E_fb_) of Cu@d‐gC_3_N_4_ was approximately −0.7 V (vs NHE), as determined from the intersection between the Mott‐Schottky plot and the baseline. In *n*‐type semiconductor photocatalysts, the conduction band generally lies 0.1 to 0.2 eV above the flat‐band potential, with this offset influenced by factors such as electron effective mass and carrier concentration. So, the conduction band potential (E_CB_) of Cu@d‐gC_3_N_4_ was approximately −0.9 V (vs NHE). Based on E_VB_ = E_CB_ + E_g_, the valence band (VB) of Cu@d‐gC_3_N_4_ was at ca. +1.49 V (vs NHE).

Later, further characterization techniques such as HAADF‐STEM (to confirm presence of Cu as single‐atom active sites), SS‐NMR (elemental composition and local chemical environment of the host matrix), XAS (further confirmation of Cu as single‐atom active site, understand the valence state and neighboring environment), XPS (surface electronic structure and oxidation state of the anchored Cu species) were performed. All these techniques provided a complete picture of the catalyst's atomic architecture, confirming the successful incorporation of single‐atom Cu centers within the defective gC₃N₄ framework and their potential role in photocatalytic reactivity.

#### 2.2.1 High‐Angle Annular Dark‐Field Scanning Transmission Electron Microscopy (HAADF‐STEM)

**Figure 3:**
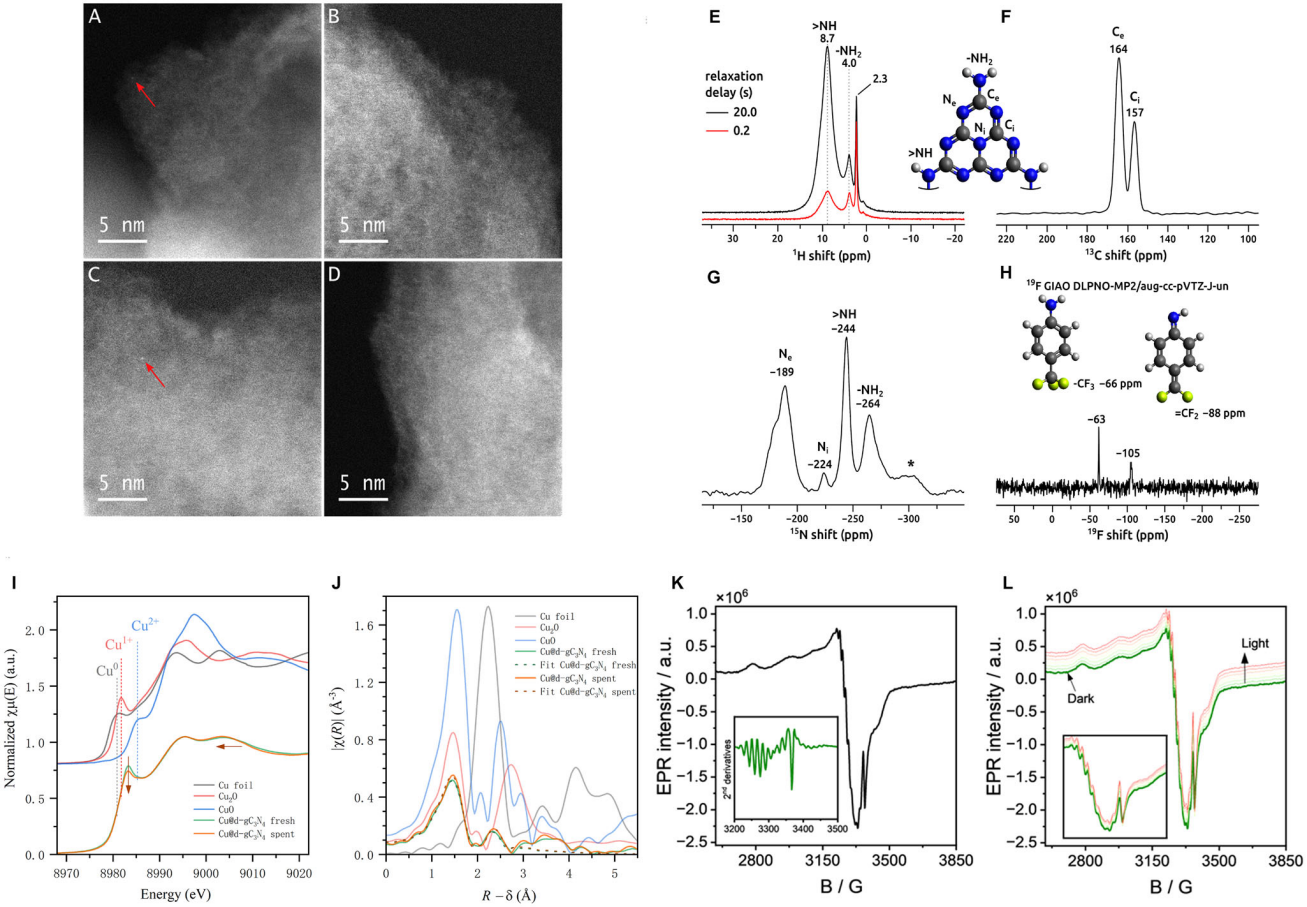
Selected STEM‐HAADF images of Cu@d‐gC_3_N_4_ before A,B) and after C,D) performing the catalytic reaction. The red arrows indicate single centers. Solid‐state ^1^H MAS E), ^13^C CPMAS F), ^15^N CPMAS G), and ^19^F MAS H) spectra collected from the Cu@d‐gC_3_N_4_ catalyst. Spinning sideband in panel G is marked with an asterisk. In the inset in panel H models of fluorinated functional groups with calculated ^19^F NMR shifts are shown. Experimental Cu *K*‐edge I) normalized XANES spectra and J) Phase‐uncorrected Fourier transform (FT) of *k^2^* – weighted EXAFS spectra (solid) and fitted spectra (dotted) for the samples. FT spectrum of the Cu foil was scaled down by a factor of 2 to facilitate visual comparison with the samples’ spectra. EPR spectra measured at 293K of (K) Cu@d‐gC_3_N_4_, with its second derivative shown in the inset, and (L) Cu@d‐gC_3_N_4_ before (green) and after irradiation with 390 nm light.

To investigate the appearance of copper (Cu) on the support, Cu@d‐gC_3_N_4_ photocatalyst (both before and after the reaction) was investigated by scanning transmission electron microscopy (STEM) coupled with energy‐dispersive X‐ray (EDX) spectroscopy. The EDX spectra of various regions of the photocatalyst before the reaction indicated that Cu was atomically dispersed over the gC_3_N_4_ support due to the weak Cu K_α_ signal (see Figure S5, Supporting Information). Because of the Z contrast between Cu and C, N in C_3_N_4_, high‐angle annular dark field (HAADF) imaging at sufficiently thin sample positions allows for the visualization of Cu. Accordingly, single Cu atomic sites are indicated in **Figure** **3**A,B. It is to be noted that along with single‐atomic metal sites, the occurrence of nanoparticle sites cannot be ruled out by STEM because of the small contents of Cu. To analyze the stability of Cu@d‐gC_3_N_4_, a similar analysis was repeated for the photocatalyst after the catalytic reaction. The HAADF‐STEM images (Figure 3C,D) and EDX spectra exhibited a similar distribution of Cu on the support, confirming the structural integrity of Cu@d‐gC_3_N_4_ toward the reaction conditions.

#### 2.2.2 Solid‐State Nuclear Magnetic Resonance (SS‐NMR)

Solid‐state NMR spectra of all NMR‐active nuclei present in the material (^1^H, ^13^C, ^15^N, and ^19^F) were collected to confirm the chemical structure of the functionalized graphitic carbon nitride catalyst. The ^1^H MAS, ^13^C CPMAS, and ^15^N CPMAS spectra (Figure 3E–H) exhibited very similar appearance and chemical shifts to those reported by us recently for related polymeric carbon nitride catalysts.^[^ ^37^, ^40^, ^41^, ^42^ ^]^ Therefore, it can be concluded that the overall chemical structure and polymerization degree in these materials is very similar. In addition to ^1^H NMR signals from the terminal (surface) ─NH_2_ groups and the bridging >NH linkers at 4.0 and 8.7 ppm, respectively, an additional minor ^1^H NMR signal at 2.3 ppm was observed, which most probably originated from hydroxyl protons. Functionalization of the graphitic carbon nitride was evidenced by the ^19^F MAS NMR spectrum shown in Figure 3H. Two observed ^19^F NMR signals at −66 and −105 ppm indicated the existence of ─CF_3_ and = CF_2_ groups in the material.^[^ ^42^, ^43^, ^44^ ^]^ To corroborate assignments of these ^19^F NMR signals, chemical shifts were calculated for both ─CF_3_ and = CF_2_ scenarios by considering respective models shown in Figure 3H. ^19^F NMR shifts for these models were calculated at the DLPNO‐MP2 level of theory using a large, uncontracted aug‐cc‐pVTZ‐J basis set.^[^ ^45^ ^]^ The obtained ^19^F NMR shift of −66 ppm for the ─CF_3_ group was very close to the signal observed at −63 ppm. This signal was also very fairly narrow, which was consistent with the rotation of the ─CF_3_ group. For the model with = CF_2_ scenario, the calculated shift of −88 ppm was relatively close to the experimental result of −105 ppm, given the broad range of chemical shifts for the ^19^F nucleus. It is therefore strongly suggested that the precursor underwent partial defluorination and was present in the material as a mixture of two structures shown in Figure 3H.

#### 2.2.3 X‐Ray Absorption Spectroscopy (XAS)

For further approval of the presence of Cu as single atom site, X‐ray absorption spectroscopy (XAS) was conducted. Upon performing the XANES and EXAFS experiments of Cu@d‐gC_3_N_4_ photocatalyst, valence state and neighboring coordination environment of Cu‐atoms in the matrix were clearly identified. In Figure 3I, the comparison of Cu K‐edge XANES spectra between photocatalyst and standard reference compounds (Cu foil, CuO and Cu_2_O) provided that the oxidation state of the Cu@d‐gC_3_N_4_ photocatalyst remained in between Cu_2_O and CuO indicating that Cu‐atoms were partially positively charged (Cu^δ+^ 1 < δ < 2).^[^ ^46^ ^]^ Next, extended X‐ray absorption fine structure (EXAFS) fitting was conducted in R‐space, after Fourier transforming the data in the k‐range of 3–12 Å⁻¹ with k¹, k^2^, and k^3^ weightings applied simultaneously (Figure 3J). The fitting was carried out over an R‐range of 1–3 Å (phase‐uncorrected), considering two scattering paths: Cu–N and Cu–C which coincide well with the Cu‐N and Cu‐C peaks for the Cu@d‐gC_3_N_4_ photocatalyst (C_32_H_16_CuN_8_) (Table S1, Supporting Information). The amplitude reduction factor (S₀^2^) was fixed at 0.867, as determined from the fit of the Cu foil reference. The reduced intensity of peak at ≈2.2 Å suggested a minor formation of Cu─Cu bond in the photocatalyst, compatible with the main presence of Cu as an isolated atom atomically dispersed on a modified g‐C_3_N_4_ matrix through Cu─N─C bonding and a minor Cu clustering.^[^ ^46^, ^47^, ^48^ ^]^ These results aligned precisely with the HAADF‐STEM observations discussed earlier and with the EPR results discussed below. It is worth mentioning that XANES spectra of the photocatalyst before reaction as well as after MF‐dehydrogenation exhibited no significant change in the local electronic and geometric structure around the absorbing atom. Furthermore, the Fourier transform (FT) of the k^2^‐weighted EXAFS oscillations also confirmed that the photocatalyst kept its coordination environment after the dehydrogenation reaction, supporting an atomically dispersed structure (Figure 3J).

#### 2.2.4 Electron Paramagnetic Resonance (EPR) Spectroscopy

In addition to all these above‐mentioned characterizations, the EPR spectrum of Cu@d‐gC_3_N_4_ (Figure 3K,L) indicated that Cu was dispersed as single atoms, exhibiting an axial signal with hyperfine structure due to interactions between the unpaired electron and the nuclear spin of copper (I = 3/2). The perpendicular component of the axial signal reveals a well‐resolved nine‐line (derivative spectrum in the inset) superhyperfine structure (SHFS), resulting from interactions between the unpaired electron and four equivalent *^14^N* nuclei (I = 1). This superhyperfine splitting suggests that each Cu atom is stabilized by coordination with four N atoms within the d‐gC_3_N_4_ matrix.^[^ ^49^ ^]^ Upon irradiation (SI, Figure 3L), the EPR signal of d‐gC_3_N_4_ increased due to a rise in the number of photoexcited electrons in the conduction band.

#### 2.2.5 X‐Ray Photoelectron Spectroscopy (XPS)

To achieve further insights into the surface composition of Cu@d‐gC_3_N_4_ X‐ray photoelectron spectroscopy (XPS) was performed. Figure S7 (Supporting Information) (see in ESI) clearly depicted XPS peaks attributing to g‐C_3_N_4_ phase as follows: (i) 286.2 eV (C in C(3)‐N), (ii) 288.3 eV (sp^2^‐bonded C atoms in N‐C = N), (iii) 289.3 eV (N = C‐O) in C 1s region, (iv) at 398.7 eV (sp^2^‐hybridized N atoms in C‐N = C), (v) 400.0 eV (bridging N atoms in N‐C(3)), (vi) 401.1 eV (N atoms in ‐NH_2_ groups) and (vii) 404.6 eV (π‐excitation) in the N 1s region.^[^ ^50^, ^51^, ^52^ ^]^A small amount of oxygen doping in the catalyst was also observed from the XPS quantification (2.0 at.%), with peaks at (i) 531.8 eV and (ii) 533.2 eV in O 1s region probably correlated with O bound to C as C = O, C‐O‐C, and/or C‐O‐H. The properly developed g‐C_3_N_4_ phase was further confirmed from the determined atomic ratio N/C = 1.1, which was a bit lower than the expected value. The Cu 2p spectrum shows a noisy peak ≈933 eV indicating the presence of a low amount of Cu on the surface of the photocatalyst.

### 2.3 Proposed Mechanism and Mechanistic Investigation

**Figure 4:**
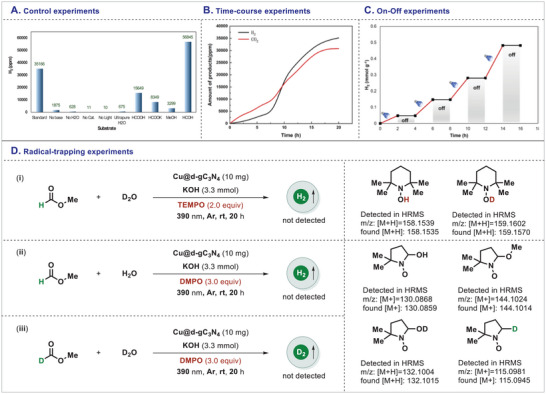
Mechanistic experiments: A) control experiments, B) time course experiments, C) light switch on‐off experiments, D) radical‐trapping experiments.

**Figure 5:**
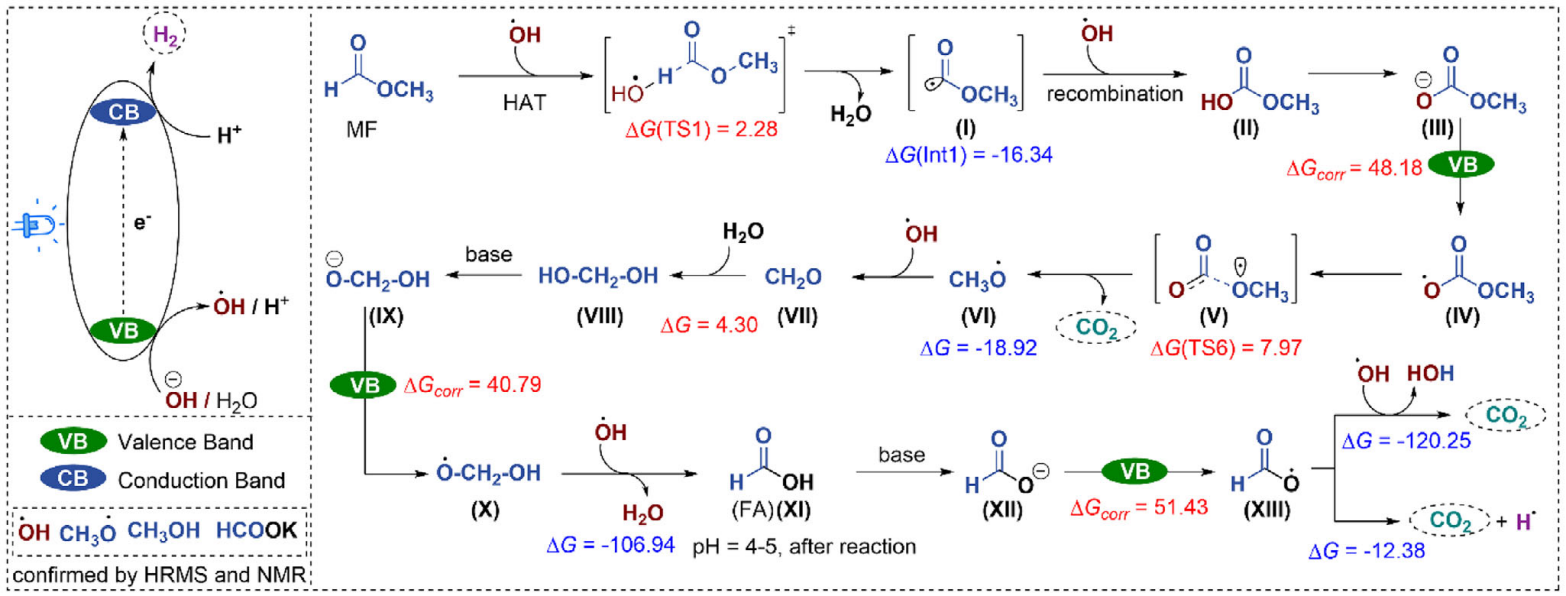
Proposed mechanism of photocatalytic transformation.

After the optimizations of the MF dehydrogenation and characterizations of the photocatalyst, we became interested in investigating the mechanism of this reaction. For this reason, a series of control experiments was conducted (**Figure** **4**A) and all the reagents (base and water) as well as the photocatalyst and the presence of light were found to be essential to achieve high reactivity and selectivity in this reaction. Furthermore, irradiation of ultrapure H_2_O in the absence of MF under the optimized reaction conditions resulted in a very low generation of H_2_. On the other hand, substituting MF by HCOOH, HCOOK, MeOH or HCHO (stabilized in methanol) under the optimized reaction conditions did not yield any significant benefit to produce H_2_. All these experiments underscored the critical role of standard reaction conditions for the efficient dehydrogenation of MF to generate green H_2_. Control experiments revealed that HCOOH, HCOOK, MeOH and HCHO likely served as key reaction intermediates (**Figure** **5**). Isotopic labeling and kinetic analyses further indicated that the primary hydrogen sources were MF and its fragmented derivatives (Figure 4A), along with contributions from H₂O explained the steam reforming process. The time‐resolved profile (Figure 4B) indicates a steady increase in the production of both H₂ and CO₂ throughout the 20‐hour duration. The first induction phase occurs for ≈8 h, succeeded by a marked increase in gas evolution, signifying the progressive activation of the catalytic system. At 20 h, H₂ attains 7.60 mmol g_cat_⁻¹ and CO₂ reaches 6.68 mmol g_cat_⁻¹, underscoring the effective and continuous reforming of methyl formate. The strong link between H₂ and CO₂ production validates the stoichiometry of the reforming pathway and affirms the system's selectivity and stability over time. Additionally, the time‐course experiments revealed that CO_2_ was released prior to hydrogen generation (Figure 4B) and this observation might be attributed to either a non‐catalytic background reaction or the hydrolysis of MF to potassium formate, leading to the initial release of CO_2_. Furthermore, the light on‐off experiment confirmed that the catalytic process underwent photo‐induced redox reaction (Figure 4C), as evidenced by the significant dropping of the reaction rate in the absence of light. In the presence of the light, the catalyst facilitated electron transfer process to drive the formation of product. Gratifyingly, its redox activity persisted through multiple cycles with light switched on which clearly emphasized its role in the electron transfer process rather than an energy transfer process. Since photocatalytic processes are mostly radical‐driven, 2,2,6,6‐tetramethylpiperidine‐1‐oxyl (TEMPO) was introduced as a radical quencher in the model reaction in the presence of D_2_O (Figure 4D‐i). D_2_O was chosen over H_2_O to exclude the minor production of H_2_ from H_2_O and to differentiate the potential radical species generated from MF and D_2_O. Without any surprise, the generation of H_2_ was completely halted, and gratifyingly, the presence of •OH/•OD or •H/•D radicals were detected through HRMS analyses. This experiment demonstrated the involvement of radical intermediates, with TEMPO functioning as a radical scavenger that effectively inhibited hydrogen generation. When TEMPO was added to a solution containing Cu/d‐gC_3_N_4_, MF, H_2_O and KOH, the three‐line EPR signal characteristic of the *N*‐centered radical of TEMPO gradually decreased over time (Figure S16C, Supporting Information), likely due to the formation of TEMPO‐OH. The generation of O_2_ was also observed simultaneously, therefore, the possibility of the formation of •OH/•OD radical was not fully excluded. After this, 5,5‐Dimethyl‐1‐pyrroline *N*‐oxide (DMPO) was added as a radical scavenger under our standard reaction conditions and both •OH and •OMe radicals were trapped which was proved by HRMS analyses (Figure 4D‐ii).

Furthermore, when DMPO was added in a mixture of d_1_‐MF (DCOOMe) and D_2_O under our standard catalytic conditions, trapping of •OH, •OD, •OMe and •D radical species was achieved (Figure 4D‐iii). These trapping experiments clearly indicated the generation of •OH from KOH/H_2_O and •H/•OMe from MF which are the key intermediates in this dehydrogenation process. The valence band maximum (VBM) energy (E_VB_) of the Cu@d‐gC_3_N_4_ photocatalyst is ≈1.50 V versus the normal hydrogen electrode (NHE). This energy level makes the oxidation of •OH/H_2_O, which requires 2.73 V versus NHE,^[^ ^38^ ^]^ highly unlikely to occur at the valence band (VB). The reaction was also investigated using in situ EPR with DMPO as a spin trap to detect short‐lived radicals. In experiments involving MF, H_2_O, and KOH, complex spectra were observed in the dark (Figure S16A, Supporting Information), indicating the formation of three DMPO‐adducts corresponding to two carbon‐centered radicals and •OH (a_N_ = a_H_ = 14.4 G) radicals. One of the DMPO‐adduct signals, with hyperfine splitting of a_N_ = 15.3 G and a_H_ = 18.3 G, is attributed to the DMPO/•COOMe adduct, while another, with hyperfine splitting of a_N_ = 15.4 G and a_H_ = 22.02 G, is characteristic of the DMPO/•COO adduct.^[^ ^53^ ^]^ Upon irradiation, the DMPO/•COOMe adduct and •OH rapidly disappeared, while the EPR signals for the DMPO/•COO adduct became dominant over time, and this should be the CO_2_•− radical anion, formed from CO_2_ as the final product getting one electron on the CB. We further confirmed the generation of these radicals through DFT. In addition, to determine the reaction order of our dehydrogenation transformation, the concentration of the base was varied and found that KOH had minimal influence on the reaction order. A similar observation was also noted when the concentration of H_2_O was altered. These experiments demonstrated that the reagents had a minimal effect on the order of the reaction and highlighted the significant role of methyl formate concentration in controlling the reaction order.

Based on our experimental studies and to further understand the reaction mechanisms (Figure 5), next we carried out detailed DFT computation (M06L‐SCRF/TZVP). This methodology had been earlier used for the light‐mediated nickel catalyzed radical allylic silylation of allyl acetates.^[^ ^54^ ^]^ At first, the reaction thermodynamics of the stoichiometric hydrolysis of MF [HCOOCH_3_ + H_2_O = HCOOH + CH_3_OH] was computed. For gas phase reaction under the standard conditions, the computed reaction was endothermic by 3.71 kcal mol^−1^, in agreement with the value [Δ_f_ *h* °(g) = 4.66 kcal mol^−1^] deduced from the standard enthalpy of formation (Δ_f_ *h* °_g_).^[^ ^55^ ^]^ The reaction was endergonic in gas phase (Δ_f_ *g* °(g) = 2.94 kcal mol^−1^). In water solution, the computed reaction was endothermic by 4.88 kcal mol^−1^, stronger than that (Δ_f_ *h* °(l) = 1.65 kcal mol^−1^) deduced from the standard enthalpy of formation, and this deviation could come from difference between molecules in their condensed forms and the solvated states in water solution. The reaction was endergonic in water solution (Δ_f_ *g* °(l) = 3.81 kcal mol^−1^). Nevertheless, all these showed that the hydrolysis of MF was not favorable thermodynamically, and only a larger amount of water could shift the reaction toward the formation of formic acid and methanol. All computational details are given in the supplementary materials.

The catalytic process is initiated by the irradiation of the semiconductor‐based photocatalyst with visible light. Upon excitation, the Cu@d‐gC_3_N_4_ photocatalyst becomes activated, resulting in the generation of positive holes in the valence band and an accumulation of excess electrons in the conduction band on its surface. Then, the catalytic cycle initiates with the reductive quenching by a single‐electron transfer (SET) from the base to the photocatalyst (PC), resulting in the generation of •OH radicals (Δ*G*_corr_ = 61.31 kcal mol^−1^). Among several possibilities (Scheme S1, Supporting Information), it is found that this transient •OH radical abstracting the C─H bond from MF to producing acyl radical species (**I**,, •CO‐OCH_3_) has the lowest Gibbs free energy barrier (TS1, 2.28 kcal mol^−1^) and is exergonic (−16.34 kcal mol^−1^). Subsequently, the recombination of •OH and (**I**) leads to the formation of methyl carbonic acid (**II**, HO‐CO‐OCH_3_) as an exergonic energy favorable step (‐96.64 kcal mol^−1^). In basic solution, methyl carbonic acid could be deprotonated to methyl carbonate (**III**, ^−^O‐CO‐OCH_3_), which could be photochemically oxidized to methyl carbonate radical (**IV**, •O‐CO‐OCH_3_, Δ*G*_corr_ = 48.18 kcal mol^−1^). The subsequent dissociation of methyl carbonate radical (**IV**) into CO_2_ and methoxy radical (**VI**, •OCH_3_) has a low Gibbs free energy barrier (TS2, 7.97 kcal mol^−1^) and is exergonic (^−1^18.92 kcal mol^−1^). At this step, one could see that the first reaction was the formal methyl formate hydrolysis with the formation of H_2_, CO_2_ and methanol [HCOOCH_3_ + H_2_O = H_2_ + CO_2_ + CH_3_OH].

The next step should be the reaction of the formed methoxy radical (**VI**). Among several possibilities for methoxy radical (Scheme S3, Supporting Information), the reaction of •OCH_3_ and •OH into formaldehyde (**VII**, CH_2_O) and H_2_O is highly exergonic (‐86.76 kcal mol^−1^) and very favored thermodynamically. It is known experimentally than formaldehyde (**VII**) could react with water to form methanediol (**VIII**, HOCH_2_OH). This reaction was computed to be slightly endergonic (4.30 kcal mol^−1^), and excess water could shift the reaction toward the formation of methanediol (**VIII**). In the basic solution, methanediol (**VIII**) could be further deprotonated by base to hydroxyl methanolate (**IX**, ^−^OCH_2_OH), which could be photochemically oxidized to the corresponding hydroxyl methoxy radical (**X**, •OCH_2_OH, Δ*G*_corr_ = 40.79 kcal mol^−1^). Further reaction of (**X**) with •OH leading to formic acid (**XI**, HCOOH), and this reaction is highly exergonic (−106.94 kcal mol^−1^). Under basic conditions, formic acid existed in the form of formate (**XII**, HCOO^−^), which could be photochemically oxidized to the corresponding radical (**XIII**, HCOO•, Δ*G*_corr_ = 51.43 kcal mol^−1^) leading to the simultaneous release of H_2_ and CO_2_. It is found that the formed radical could either dissociate thermodynamically favorable to CO_2_ and hydrogen atom (‐12.38 kcal mol^−1^) or reacted with •OH to CO_2_ and H_2_O, and the last step was very favorable thermodynamically (‐120.25 kcal mol^−1^). In the photocatalyst system, the oxidation of formic acid or H_2_O by photogenerated holes produced protons. These protons were subsequently reduced by photogenerated electrons in the conduction band, leading to the formation of hydrogen. However, the possibility of hydrogen generation through radical‐radical coupling of the formed •H radicals could not be excluded. We further calculated the quantum yield of this process to assess whether the reaction followed a radical chain mechanism (see the Supporting Information for details), and the low quantum yield (Φ = 0.103) indicated that a radical chain process was unlikely to be involved. While the observed quantum yield is modest, the system's high selectivity, operational stability, and ability to function under ambient conditions highlight its promise for LOHC applications. We anticipate that further catalyst and system optimization will lead to improved efficiency.

## 3 Conclusion

In conclusion, we present a Cu‐based atomically dispersed photocatalyst for the generation of green hydrogen from methyl formate (MF). Under optimized reaction conditions, hydrogen production from MF occurs at room temperature and eliminates possible limitations (requirement of elevated temperatures, metal catalysts, or complex reaction setups) of hydrogen generation from traditional hydrogen storage chemicals such as formic acid and methanol. Notably, our MF dehydrogenation technique represents the first instance of photocatalytic green hydrogen generation from this hydrogen storage material. This photocatalytic system is particularly promising for further scale‐up and practical applications due to its purely heterogeneous nature and high stability. In addition, this system shows high reactivity, selectively producing 7.5 mmol g_cat_^−1^ of hydrogen (nothing else than H_2_ and CO_2_). After extensive mechanistic studies, and characterizations of our photocatalyst, we propose a radical reaction pathway that deviates from the traditionally known thermal mechanisms reported in the literature. Based on the results presented here, we anticipate that future research will allow the design of new catalytic systems – whether thermochemical, photochemical, or electrochemical – for hydrogen generation from methyl formate and make this technology a reality.

## 4 Experimental Section

### Synthetic Procedure of the Photocatalyst

The single‐atom metal heterogeneous photocatalyst Cu@d‐gC_3_N_4_ was prepared through a two‐step process involving successive impregnation and calcination in the absence of a template. In a typical synthesis, dicyandiamide (DCDA, 9 g, 107 mmol) and 2‐amino‐5‐(trifluoromethyl) benzonitrile (0.150 g, 0.8 mmol, 1.6 wt.%) were homogeneously mixed with Cu(NO_3_)_2_•3H_2_O (0.019 g, 0.08 mmol, 0.2 wt.%, corresponding to a Cu loading of 0.055 wt.%). The mixture was initially stirred in water (45 mL) at 95 °C for 1 h in a closed container, and subsequently dried at 100–110 °C after unsealing to remove water. The dried product was then ground in an agate mortar and loaded into a stainless‐steel chamber. The chamber was heated to 550 °C in a GERO Carbolite tube furnace for 244 min under an aerobic atmosphere. The temperature was maintained for 4 h, followed by cooling to room temperature over a period of 6 h. The Cu‐modified gC_3_N_4_ catalyst was hereafter denoted as Cu@d‐gC_3_N_4_. After calcination, the resulting catalyst weighed ≈2.9–3.3 g, corresponding to a yield of ≈33%.

### General Procedure for Dehydrogenation of Methyl formate (MF) (GP‐1)

All experiments were conducted under an inert atmosphere of Ar or N_2_ using standard Schlenk techniques. A Cu catalyst and a defined amount of base were added to an oven‐dried Schlenk tube, followed by the addition of MF and H_2_O via syringe. The reaction mixture was irradiated with 390 nm light. Upon completion of the reaction, the pressure was carefully released at room temperature (25 °C). The evolved gas was collected using a manual burette for volumetric measurement, and its composition was analyzed by GC. All experiments were performed at least twice, and the average gas volumes and compositions are reported with standard deviations < 5%.

## Conflict of Interest

The authors declare no conflict of interest.

## Supporting information



Supporting Information

## Data Availability

The data that support the findings of this study are available on request from the corresponding author. The data are not publicly available due to privacy or ethical restrictions.
