# Supplementary material for: Photocatalytic Aqueous Reforming of Methyl Formate
Source: Adv Mater. 2025 Jul 11;37(39):2509890. doi: 10.1002/adma.202509890 (PMC12506610; doi:10.1002/adma.202509890)
Supplement: Supplementary file 1 — Supporting Information [file ADMA-37-2509890-s001.docx]

***Supporting Information***

Photocatalytic Aqueous Reforming of Methyl Formate

Dongxu Zuo,^1†^ Suman Pradhan,^1†^ Manami Banerjee,^1^ Nils Rockstroh,^2^ Stephan Bartling,^2^ Abdallah I.M. Rabee,^3,2^ Xinxin Tian,^4,2^ Alina Skorynina,^5^ Aleksander Jaworski,^6^ Laura Simonelli,^5^ Jabor Rabeah,^7,2^ Haijun Jiao,^2^* Matthias Beller,^2^* and Shoubhik Das^1^*

^1^Department of Chemistry, University of Bayreuth, Universitatsstraße 30, 95447, Bayreuth, Germany

^2^Leibniz-Institut für Katalyse e.V. (LIKAT Rostock), Albert-Einstein-Str. 29a, 18059 Rostock, Germany.

^3^Chemistry Department, Faculty of Science, Minia University, El-Minia, 61519, Egypt

^4^Institute of Molecular Science, Key Laboratory of Chemical Biology and Molecular Engineering of Ministry of Education, Shanxi University, Taiyuan 030006, P. R. China

^5^CLAESS Beamline, ALBA Synchrotron Light Facility, Carrer de la Llum 2−26, Barcelona, Cerdanyola del Vallès 08290, Spain

^6^Department of Chemistry, Stockholm University, Stockholm 10691, Sweden

^7^State Key Laboratory of Low Carbon Catalysis and Carbon Dioxide Utilization, Lanzhou Institute of Chemical Physics (LICP), Chinese Academy of Sciences, Lanzhou 730000, P. R. China

*E-mail: [Haijun.Jiao@catalysis.de](mailto:Haijun.Jiao@catalysis.de), [Matthias.Beller@catalysis.de](mailto:Matthias.Beller@catalysis.de), [Shoubhik.Das@uni-bayreuth.de](mailto:Shoubhik.Das@uni-bayreuth.de)

† These authors contributed equally to this work.

**Content**

[1. General Information 3](#_Toc201738446)

[1.1 Chemicals and solvents 3](#_Toc201738447)

[1.2 Analytical methods 3](#_Toc201738448)

[2. Synthesis of Catalysts 4](#_Toc201738449)

[2.1 Synthesis of Cu dispersed modified gC_3_N_4_ catalysts 4](#_Toc201738450)

[2.2 Synthesis of normal gC_3_N_4_, d-gC_3_N_4_ and Cu@gC_3_N_4_ 5](#_Toc201738451)

[3. Characterization of the Photocatalyst 6](#_Toc201738452)

[3.1 TEM and EDX 6](#_Toc201738453)

[3.2 X-ray photoelectron spectroscopy (XPS) 7](#_Toc201738454)

[3.3 Solid-state nuclear magnetic resonance (ssNMR) 8](#_Toc201738455)

[3.4 Electron paramagnetic resonance (EPR) characterization 9](#_Toc201738456)

[3.5 X-ray absorption spectroscopy (XANEX and EXAFS) 9](#_Toc201738457)

[3.6 Electronic band positions measurement 10](#_Toc201738458)

[4. Experimental Procedure 13](#_Toc201738459)

[4.1 General procedure 13](#_Toc201738460)

[4.2 Calculation of gas volume and catalytic performance 13](#_Toc201738461)

[4.3 Optimization of reaction conditions 15](#_Toc201738462)

[4.4 Apparent quantum yield (AQY) measurement 19](#_Toc201738463)

[4.5 Comparison of dehydrogenation in different substances 20](#_Toc201738464)

[4.6 Time course experiment 20](#_Toc201738465)

[4.7 Conversion of MF dehydrogenation reaction 22](#_Toc201738466)

[4.8 Long-term reaction experiments 22](#_Toc201738467)

[4.9 Sunlight-driven dehydrogenation of MF 23](#_Toc201738468)

[4.10 Scale-up reaction of MF dehydrogenation 24](#_Toc201738469)

[4.11 Light on-off experiments 24](#_Toc201738470)

[4.12 Catalyst recycling 25](#_Toc201738471)

[4.13 Quenching experiments 26](#_Toc201738472)

[4.14 Reaction rate determination 26](#_Toc201738473)

[4.15 Electron paramagnetic resonance (EPR) studies 27](#_Toc201738474)

[4.16 NMR spectra of the product and reaction intermediates 28](#_Toc201738475)

[4.17 Gas Chromatogram of the collected reaction gas mixture 30](#_Toc201738476)

[4.18 Intermediate detection in aqueous reforming of methyl formate 31](#_Toc201738477)

[5. DFT Calculations 32](#_Toc201738478)

[5.1 Computational methodology 32](#_Toc201738479)

[5.2 Benchmark testing 33](#_Toc201738480)

[5.3 DFT study on the dehydrogenation of MF 33](#_Toc201738481)

[6. Reference 46](#_Toc201738482)

# 1. General Information

## 1.1 Chemicals and solvents

Commercial reagents were used without further purification and all reactions were carried out under argon atmosphere using Schlenk techniques. H_2_O was deionized water (DI H_2_O). 5,5-Dimethyl-1-Pyrrolin N-Oxid (DMPO), Methyl formate, KOH, Cu(NO_3_)_2_•3H_2_O, DCO(O)D were purchased from Thermo Fisher Scientific. Dicyandiamide, 2-amino-5-(trifluoromethyl) benzonitrile, 2,2,6,6-Tetramethyl-1-oxylpiperidine (TEMPO) were purchased from BLD. D_2_O, CD_3_OD, acteone-d_6_ were purchased from Deutero GmbH. HCOOCD_3_ was purchased from Sigma Aldrich. Mesitylene was purchased from TCI.

## 1.2 Analytical methods

### 1.2.1 Nuclear magnetic resonance (NMR)

NMR spectra were recorded on a Bruker Avance III HD 500 spectrometer operating at 500 MHz and 126 MHz for ^1^H and ^13^C acquisitions, respectively. Chemical shifts are reported in ppm with the solvent residual peak as the internal standard. NMR spectra were calibrated using the solvent residual signals (D_2_O: δ ^1^H = 4.79; Acetone-d6: δ ^1^H = 2.05, δ ^13^C = 29.80). Data is reported as follows: s = singlet, d = doublet, t = triplet, q = quartet, m = multiplet, bs = broad singlet; coupling constants in Hz; integration. ^13^C NMR spectra were recorded with complete proton decoupling.

### 1.2.2 Gas chromatography (GC)

The content of H_2_, CO_2_, and CO in the gas phase was analyzed using a GC (Nexis GC-2030 system), with He serving as the carrier gas in both cases.

### 1.2.3 High resolution mass spectrometry (HRMS)

The samples were prepared by dissolving 0.1**–**5 mg of the product in acetone-d_6_ and CD_3_OD and further diluted to a concentration of 10^-5^**–**10^-6^ M with 50% methanol (or acetonitrile)/50% H_2_O/0.1% formic acid.

### 1.2.4 Setup of the reaction system

Kessil lamps (390 nm) were purchased from Laser 2000 (UK) Ltd. The light intensity was measured to be 0.10 W/cm² using an Ophir StarLite power meter with a 3A probe head, with the distance between the tube and the light source maintained at approximately 3-4 cm. To maintain the reaction temperature within 20–30 °C, either a water bath combined with a single fan or two commercially available fans were used for cooling.


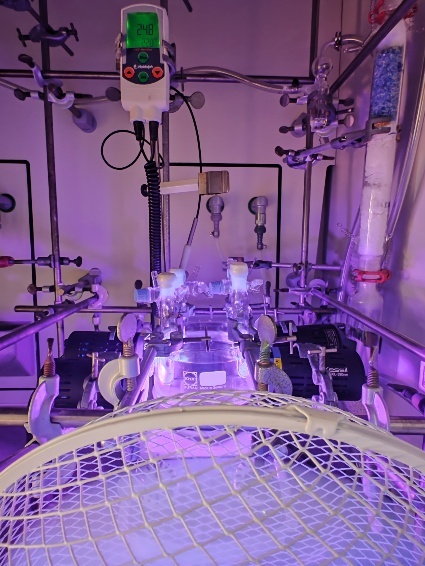


Figure S1. Setup of the reaction system.

### 1.2.5 Electron paramagnetic resonance (EPR)

EPR spectra were recorded on an X-band Bruker EMX CW-micro EPR spectrometer equipped with an ER4119HS high-sensitivity resonator using a microwave frequency of ν ≈ 9.7 GHz, a microwave power of 6.3 mW, a modulation frequency of 100 kHz and a modulation amplitude up to 5 G, a scanning number of 1 and sweeping time of 30 s. The hν = gβB_0_ equation was used to calculate g values with ν and B_0_ being the microwave frequency and resonance field, respectively. 2,2-Diphenyl-1-picrylhydrazyl (DPPH) was used as a standard (g = 2.0036 ± 0.0004) for calibration of the g value.

# 2. Synthesis of Catalysts

## 2.1 Synthesis of Cu dispersed modified gC_3_N_4_ catalysts


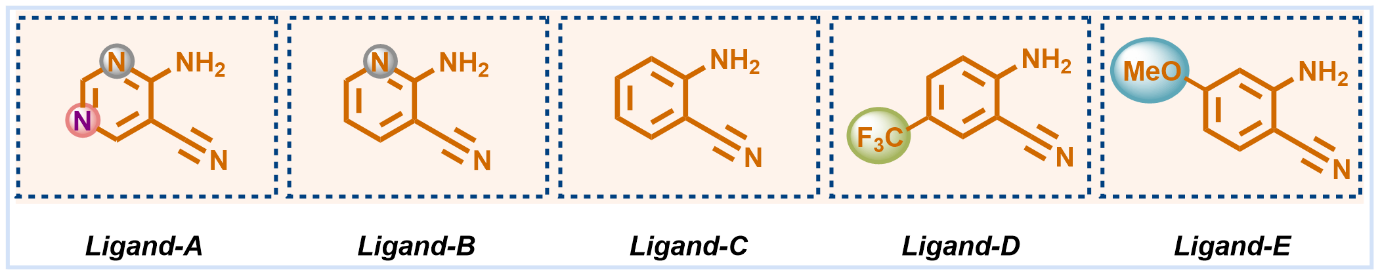


**Procedure for the synthesis of catalysts** **Cu@a-gC_3_N_4_, Cu@b-gC_3_N_4_, Cu@c-gC_3_N_4_, Cu@d-gC_3_N_4_, Cu@e-gC_3_N_4_ *(synthesis of Cu@d-******gC_3_N_4_)*:**


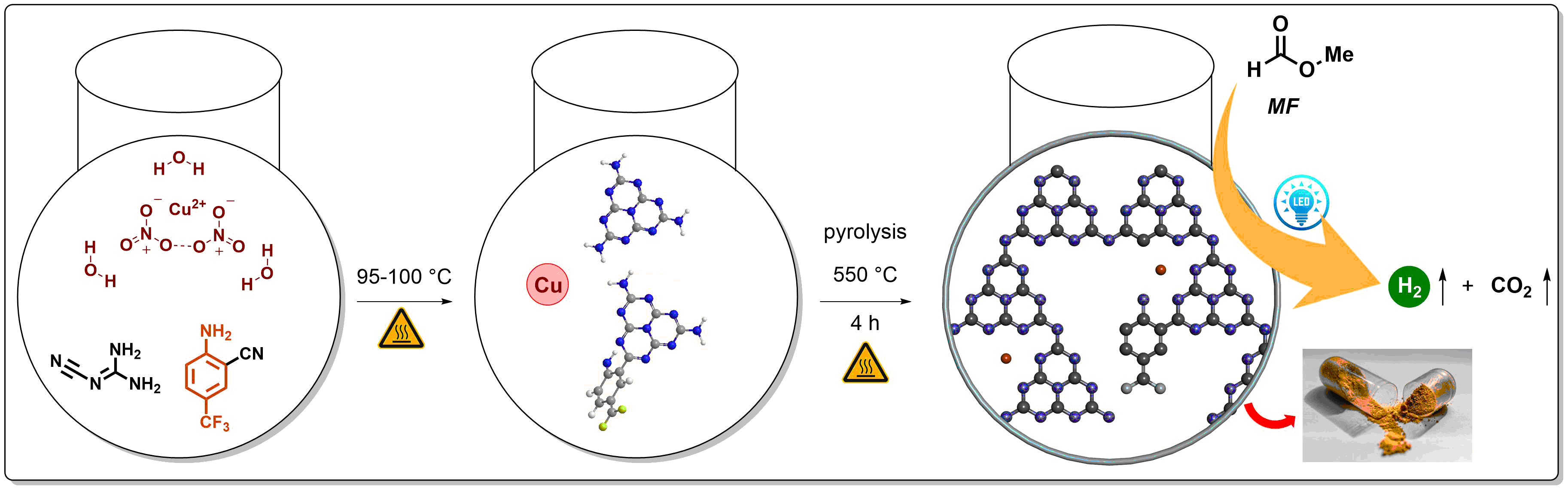


Figure S2. The preparation procedure of the single Cu atom photocatalyst (Cu**–**SAPC). Dashed bonds in the product refer to the weak interaction between N and Cu atoms.

The single-atom metal heterogeneous photocatalyst Cu@d-gC_3_N_4_ was prepared through a two-step process involving successive impregnation and calcination in the absence of a template. In a typical synthesis, dicyandiamide (DCDA, 9 g, 107 mmol) and 2-amino-5-(trifluoromethyl) benzonitrile (0.150 g, 0.8 mmol, 1.6 wt%) were homogeneously mixed with Cu(NO_3_)_2_•3H_2_O (0.019 g, 0.08 mmol, 0.2 wt%, corresponding to a Cu loading of 0.055 wt%). The mixture was initially stirred in water (45 mL) at 95 °C for 1 hour in a closed container, and subsequently dried at 100–110 °C after unsealing to remove water. The dried product was then ground in an agate mortar and loaded into a stainless-steel chamber. The chamber was heated to 550 °C in a GERO Carbolite tube furnace for 244 minutes under an aerobic atmosphere. The temperature was maintained for 4 hours, followed by cooling to room temperature over a period of 6 hours. The Cu-modified gC_3_N_4_ catalyst was hereafter denoted as Cu@d-gC_3_N_4_. After calcination, the resulting catalyst weighed approximately 2.9**–**3.3 g, corresponding to a yield of about 33%.

## 2.2 Synthesis of normal gC_3_N_4_, d-gC_3_N_4_ and Cu@gC_3_N_4_


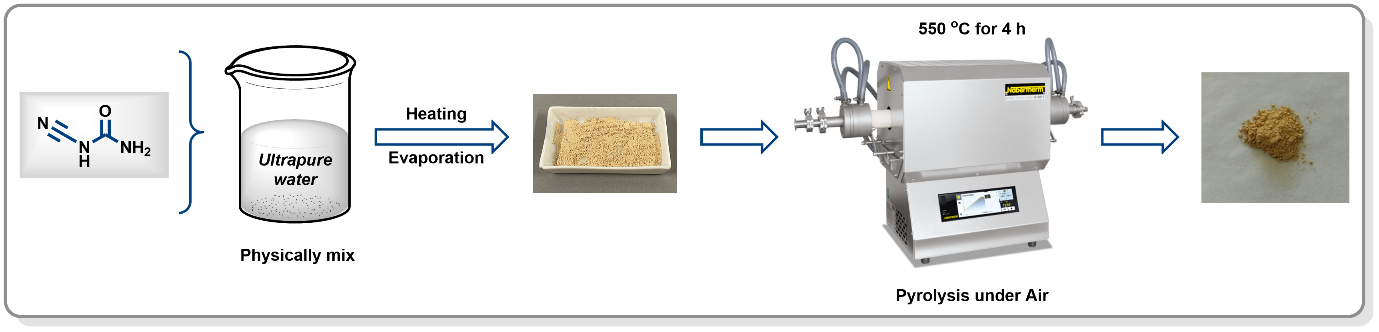


Figure S3. The preparation procedure of the normal gC_3_N_4_.

***Normal gC_3_N_4_:*** The procedure followed the same synthetic method as that used for the Cu@d-gC_3_N_4_ catalyst, except without the addition of metal precursors and 2-amino-5-(trifluoromethyl)benzonitrile.


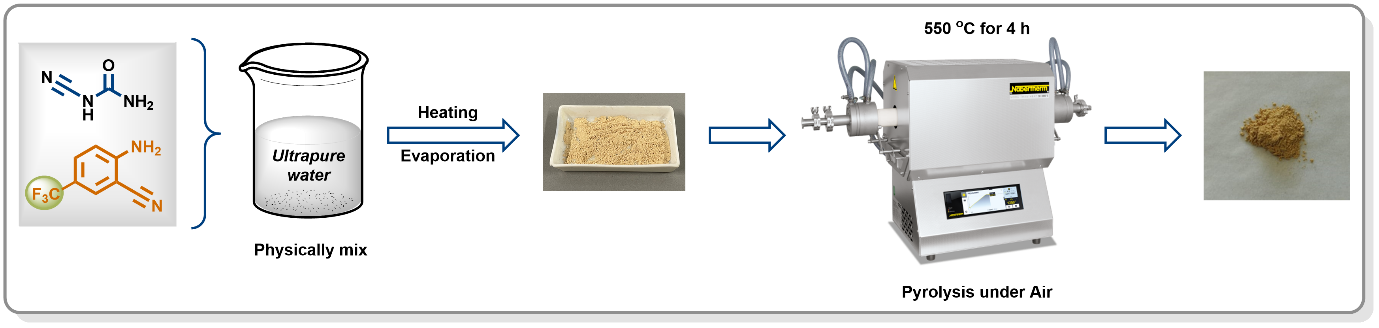


Figure S4. The preparation procedure of the d-gC_3_N_4_.

***d-gC_3_N_4_*:** The 2-amino-5-(trifluoromethyl) benzonitrile substituted g-C_3_N_4_ catalyst was named d-gC_3_N_4_, following the same synthetic procedure as the Cu@d-gC_3_N_4_ catalyst, but without the addition of metal precursors.


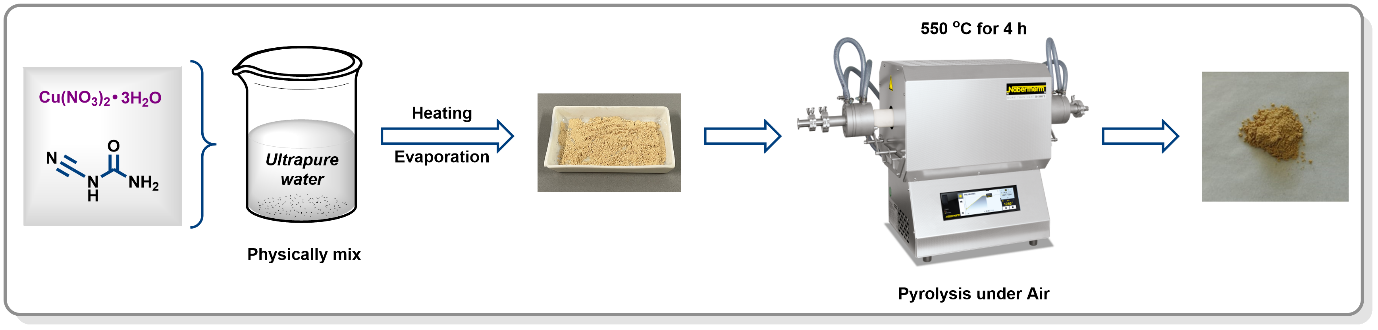


Figure S5. The preparation procedure of the Cu@gC_3_N_4_.

***Cu@gC_3_N_4_:*** The meta-doped g-C_3_N_4_ catalyst was named Cu@gC_3_N_4_, which followed the same synthetic recipe as the Cu@d-gC_3_N_4_ catalyst, but without adding 2-amino-5-(trifluoromethyl) benzonitrile.

# 3. Characterization of the Photocatalyst

## 3.1 TEM and EDX

Scanning Transmission Electron Microscopy (STEM) measurements were carried out on a probe aberration-corrected JEM-ARM200F (JEOL, Corrector: CEOS) at an operation voltage of 200 kV. The microscope is equipped with a JED-2300 (JEOL) energy-dispersive X-ray (EDX) spectrometer having a silicon drift detector (dry SD60GV). High-Angle Annular Dark Field (HAADF) and Annular Bright Field (ABF) detectors were used for STEM imaging. The solid samples were deposited onto a holey carbon supported Cu grid (mesh 300) without any pre-treatment and subsequently transferred to the microscope. The results are shown in manuscript and **Figure S5–S6**.


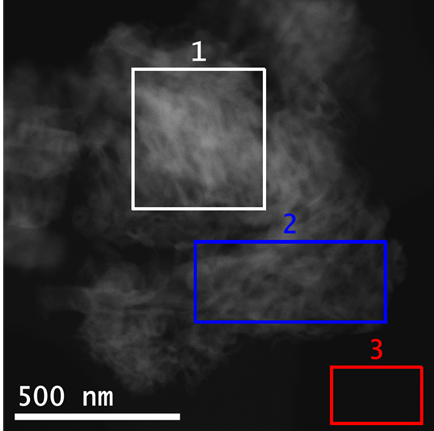

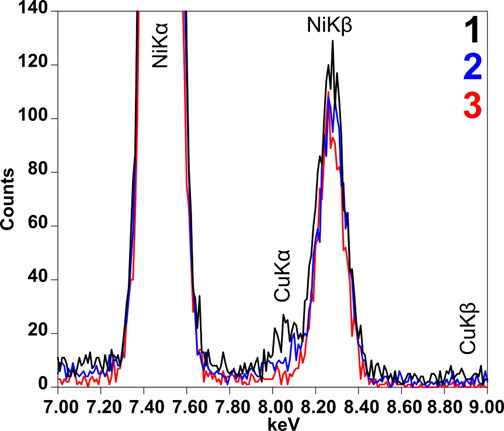


Figure S6. Selected EDX spectra of Cu@d-gC_3_N_4_ before the catalytic reaction (right) of the regions highlighted by rectangles in the STEM-HAADF image (left). Spectrum 1 shows clearly a small Cu Kα signal.


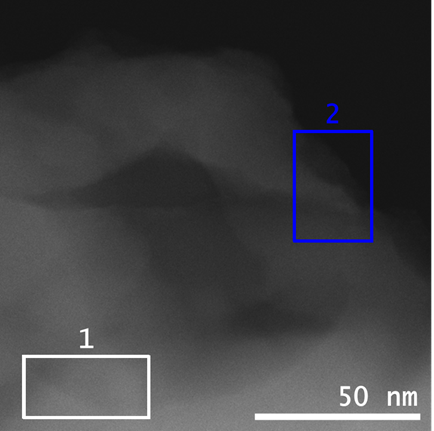

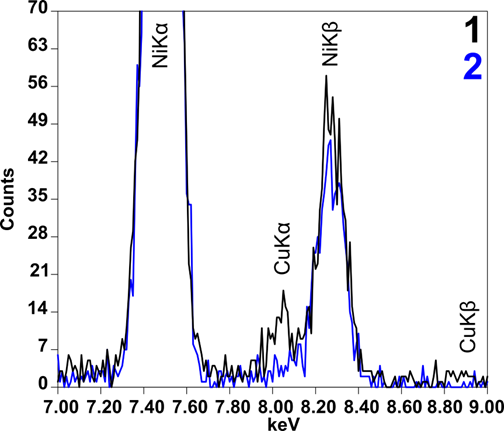


Figure S7. Selected EDX spectra of Cu@d-gC_3_N_4_ after the catalytic reaction (right) of the regions highlighted by rectangles in the STEM-HAADF image (left). Spectrum 1 shows clearly a small Cu Kα signal.

## 3.2 X-ray photoelectron spectroscopy (XPS)

The XPS (X-ray Photoelectron Spectroscopy) measurements were performed on an ESCALAB 220iXL (Thermo Fisher Scientific) with monochromated Al Kα radiation (E = 1486.6 eV). Samples are prepared on a stainless-steel holder with conductive double-sided adhesive carbon tape. The measurements are performed with charge compensation using a flood electron system combining low energy electrons and Ar^+^ ions (p_Ar_ = 1×10^-7^ mbar). The electron binding energies are referenced to the C 1s core level of carbon at 284.8 eV (C-C and C-H bonds). For quantitative analysis, the peaks were deconvoluted with Gaussian-Lorentzian curves using the software Unifit 2023. The peak areas were normalized by the transmission function of the spectrometer and the element specific sensitivity factor of Scofield.

The result is shown in **Figure S8**. A small amount of oxygen doping in the catalyst was also observed from the XPS quantification (2.0 at.%), with peaks at (i) 531.8 eV and (ii) 533. 2 eV in O 1s region probably correlated with O bound to C as C=O, C-O-C, and/or C-OH. The properly developed g-C_3_N_4_ phase was further confirmed from the determined atomic ratio N/C = 1.1, which was a bit lower than the expected value. The Cu 2p spectrum shows a noisy peak around 933 eV indicating the presence of a low amount of Cu on the surface of the photocatalyst. Other results and discussions are as shown in the manuscript.


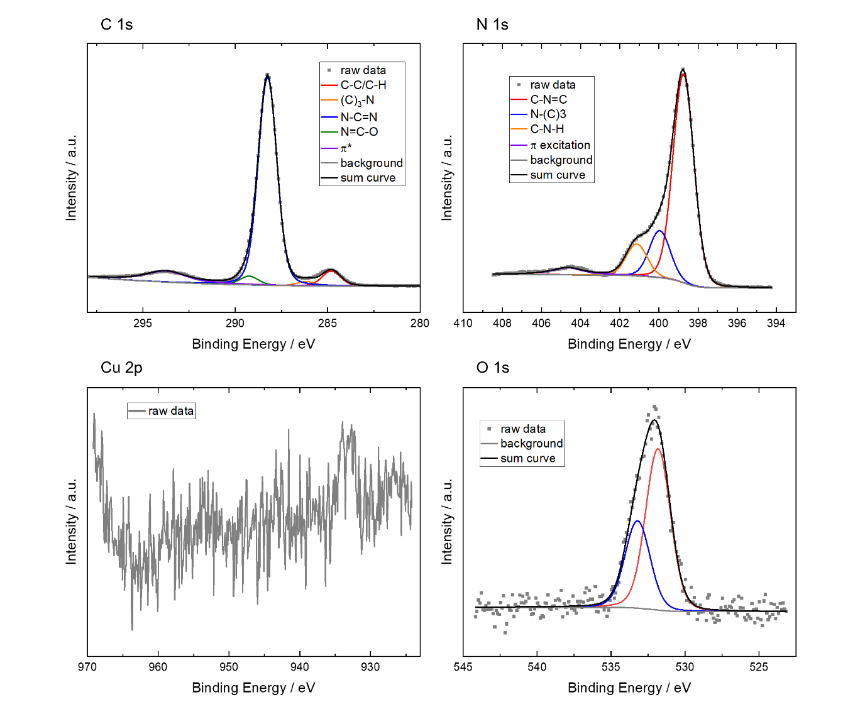


Figure S8. XPS C 1s, N 1s, Cu 2p and O 1s spectra of Cu@d-gC_3_N_4_.

## 3.3 Solid-state nuclear magnetic resonance (ssNMR)

Solid-state magic-angle-spinning (MAS) ^1^H and ^19^F NMR spectra, as well as cross-polarization ^1^H-^15^N CPMAS NMR spectra were collected at a magnetic field of 14.1 T with a Bruker Avance-III spectrometer. The ^1^H and ^19^F MAS NMR spectra were acquired using a 1.3 mm probehead and a 60 kHz MAS rate. These acquisitions involved a use of a rotor-synchronized, double-adiabatic spin-echo sequence with a 90° excitation pulse of 1.25 µs (2.50 for ^19^F) followed by a pair of 50.0 µs tanh/tan short high-power adiabatic pulses (SHAPs) with 5 MHz frequency sweep. All pulses operated at the nutation frequency of 200 kHz (100 kHz for ^19^F). 64 (16384 for ^19^F) signal transients were acquired using a relaxation delay of 20 s (5 s for ^19^F). ^1^H-^15^N CPMAS NMR spectra were recorded using a 7 mm probehead with a 7 kHz MAS rate and 65 kHz spinal64 proton decoupling. For ^1^H-^15^N CPMAS acquisition Hartmann-Hahn matched radiofrequency fields were applied for a contact interval of 5 ms and 32768 signal transients were collected using a relaxation delay of 2 s. The ^1^H-^13^C CPMAS spectrum was collected at a magnetic field of 9.4 T with a Bruker Avance-III spectrometer using a 4 mm probehead and a 14 kHz MAS rate, and involved a contact interval of 1.5 ms, and 1024 scans collected with a relaxation delay of 2 s. Chemical shifts are reported with respect to TMS (^1^H, ^13^C), nitromethane (^15^N), and trichlorofluoromethane (^19^F). Calculations of ^19^F NMR shifts were performed with the ORCA code ^[1-2]^. Geometry optimizations of the models and the subsequent (GIAO) NMR shifts calculations were performed at the revPBE-D4/pcseg-2 and DLPNO-MP2/aug-cc-pVTZ-J-un levels of theory, respectively. The CFCl_3_ molecule treated at the same level of theory was used as a ^19^F NMR shift reference.

## 3.4 Electron paramagnetic resonance (EPR) characterization

For solid state EPR, 10 mg of Cu@d-gC_3_N_4_ was placed in a normal EPR tube and then the tube was filled with Ar and sealed with a septum. Subsequently, the tube was placed into the EPR cavity, and the spectra recorded RT, both in the dark and after irradiation with 390 nm light. The results and discussions are shown in the manuscript.

## 3.5 X-ray absorption spectroscopy (XANEX and EXAFS)

Cu *K*-edge X-ray absorption spectra (XAS) were recorded at the CLÆSS^[3]^ beamline of the ALBA synchrotron (proposal #2024108916), using a double-crystal Si(111) monochromator. The X-ray beam was focused to a spot size of 200 × 100 µm² (H × V). XAS spectra were collected at room temperature under vacuum conditions in transmission mode for the standard reference compounds (Cu foil, CuO and Cu_2_O) and in fluorescence mode for the samples using 4-channel silicon drift detector (SDD). The samples were prepared as 5 mm pellets of pure material.

XAS data analysis was performed using the ATHENA and ARTEMIS software of the DEMETER^[4]^ package. Extended X-ray absorption fine structure (EXAFS) fitting was conducted in R-space, after Fourier transforming the data in the k-range of 3–12 Å⁻¹ with k¹, k², and k³ weightings applied simultaneously. The fitting was carried out over an R-range of 1–3 Å (phase-uncorrected), considering two scattering paths: Cu–N and Cu–C. The amplitude reduction factor (S₀²) was fixed at 0.867, as determined from the fit of the Cu foil reference, The results and discussions are shown in the manuscript and **Table S1.**

Table S1. EXAFS fitting parameters at the Cu K-edge

| **Sample** | **Scattering path** | **C. N.** | **Δ*E* (eV)** | **R (Å)** | **σ^2^** | **R-factor** |
| --- | --- | --- | --- | --- | --- | --- |
| Cu@C_3_N_4_ fresh | Cu–N | 3.32 ± 0.74 | 2.47 ± 2.63 | 1.92 ± 0.05 | 0.010 ± 0.003 | 0.012 |
|  | Cu–C | 2.42 ± 1.97 |  | 2.78 ± 0.12 | 0.007 ± 0.008 |  |
| Cu@C_3_N_4_ spent | Cu–N | 3.10 ± 0.73 | 2.58 ± 2.89 | 1.92 ± 0.05 | 0.009 ± 0.003 | 0.019 |
|  | Cu–C | 2.41 ± 1.82 |  | 2.82 ± 0.16 | 0.006 ± 0.007 |  |

## 3.6 Electronic band positions measurement

### 3.6.1 UV-Visible-DRS spectroscopy

The solid UV–Vis absorption curve (**Figure S9A**) was obtained from diffuse reflectance spectra (DRS) measured on a Shimadzu UV-3101PC spectrophotometer. BaSO_4_ was used as the reflectance standard. Photoluminescence experiments were conducted in solid-state on an Edinburgh FLSP920 spectrofluorometer, with a photomultiplier detector operating in the 200-900 nm wavelength range at room temperature.

This method is based on the formula proposed by Tauc, Davis, and Mott, commonly known as the Tauc plot.

$$\begin{aligned} \left( \alpha hv \right)^{\frac{1}{n}} =A\left( hv-E_{g} \right)\#\left( 1 \right) \end{aligned}$$

where hv=hc/λ. Here, α is the absorption coefficient, h is Planck's constant, v is the photon frequency, c is the speed of light, and λ is the wavelength of light. Eg is the bandgap energy. The exponent n depends on the type of semiconductor: for direct bandgap semiconductors, n = 1/2; for indirect bandgap semiconductors, n = 2. A is a parameter dependent on the transmission probability.

Additionally, the band gap energy is usually determined from diﬀuse reﬂectance spectra. For UV-Vis diffuse reflectance spectroscopy (DRS) to measure the bandgap, reflectance (R) is typically measured. The Kubelka-Munk function F(R∞) based on Kubelka-Munk theory is then used, which relates reflectance to absorbance and is given by:

$$\begin{aligned} F\left( R_{\infty} \right)=\frac{K}{S} =\frac{\left( 1-R_{\infty} \right)^{2}}{2R_{\infty}}\#\left( 2 \right) \end{aligned}$$

where R_∞ = R_sample_/R_standard_ is the reﬂectance of an inﬁnitely thick specimen, while K and S are the absorption and scattering coeﬃcients, respectively. Substitute F(R∞) for α in equation 1 yields the form equation 3.

$$\begin{aligned} \left( F\left( R_{\infty} \right)⸱hv \right)^{\frac{1}{n}} =A\left( hv-E_{g} \right)\#\left( 3 \right) \end{aligned}$$

Then plot (F(R_∞_)•hv)^1/n^ versus hv (**Figure S9B**)**.** The linear portion of the plot is then extrapolated to the horizontal axis, where the intercept gives the bandgap energy (Eg).


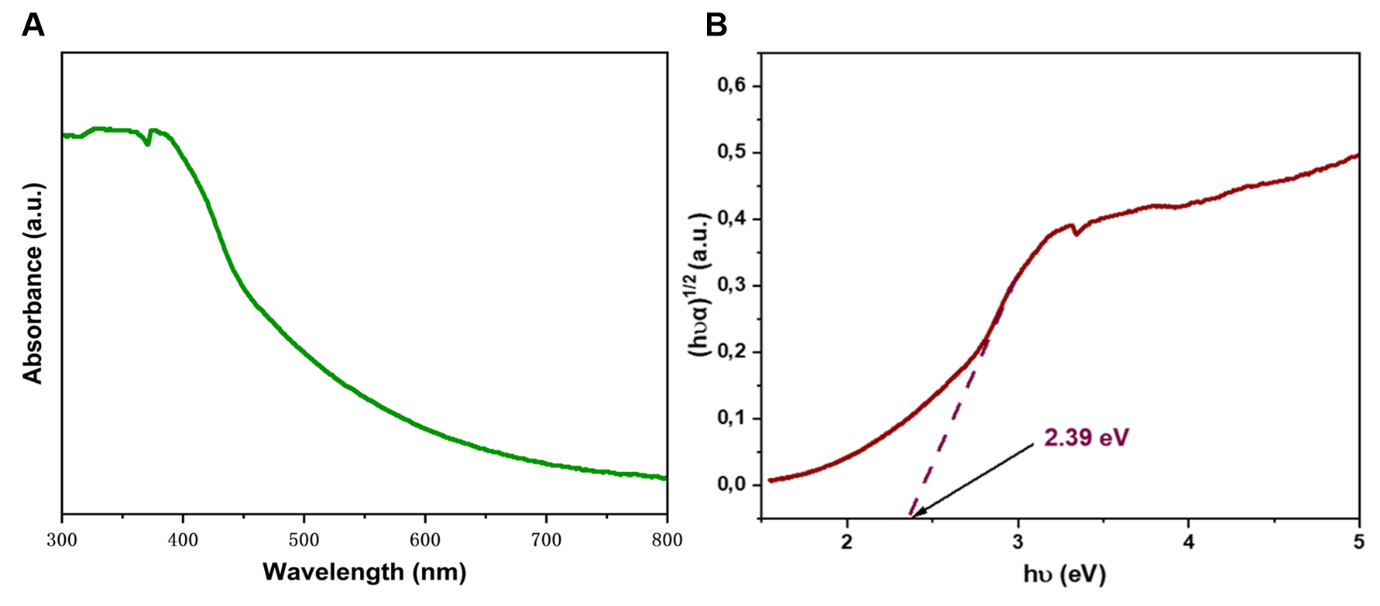


**Figure S9.** (A) UV−vis absorption spectra; (B) UV-vis diffuse reflectance spectra (DRS) plot.

### 3.6.2 Mott-Schottky (MS) measurements

The Mott-Schottky experiments were conducted employing a three-electrode configuration cell. The working electrode consisted of a thin film obtained through the drop-coating method, while the counter electrode comprised a Pt-foil electrode, and the reference electrode utilized an Ag/AgCl/3 M KCl electrode. The thin film electrodes were prepared by combining 5 mg of the sample with a solution composed of 50 μL ethanol and 10 μL of 5 wt% Nafion solution, followed by sonication for 1 hour. Subsequently, 15 μL of the resulting mixture was pipetted onto a FTO glass surface to create 1 × 1 cm^2^ coating films, which were then dried at room temperature and further subjected to overnight drying at 40 °C in a vacuum oven. The electrolyte solution employed was 0.5 M Na_2_SO_4_ (with a measured pH of 7.47), degassed with N_2_. Measurements were carried out using a Metrohm Autolab potentiostat-galvanostat PGSTAT204 workstation in the absence of light, with an AC amplitude of 5 mV and frequencies of 400 Hz, 600 Hz, and 1000 Hz.

Then, to further clarify the electronic structure, electrochemical flat-band potential measurements were conducted. Typically, flat-band potential values are derived from the Mott-Schottky equation.

$$\begin{aligned} \frac{1}{C^{2}}=\frac{2}{\varepsilon\varepsilon_{0}N_{D}}(E-E_{fb}-\frac{K_{B}T}{q})\#(4) \#\# \end{aligned}$$

Here, C represents the space charge capacitance, N_D_ denotes the donor density, and ε, ε_0_ are the dielectric constants of the film electrode and free space, respectively. E refers to the applied potential, E_fb_ is the flat-band potential, K_B_ is Boltzmann’s constant, T is the temperature, and q is the electronic charge. The flat-band potential E_fb_ can be obtained by extrapolating the plot to where 1/C^2^ = 0. The result is shown in **Figure S10**.


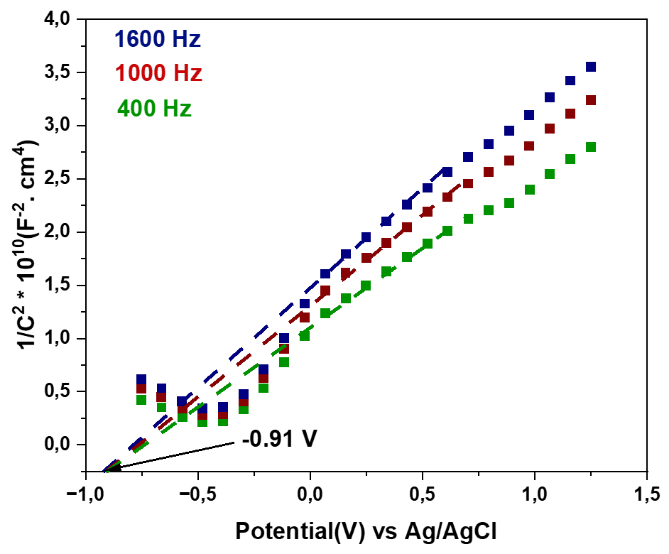


Figure S10. Mott−Schottky plots of bulk Cu@d-gC_3_N_4_.

# 4. Experimental Procedure

## 4.1 General procedure

An oven dried 25 mL Schlenk tube containing a magnetic stir bar was charged with 10 mg catalyst (0.2 wt% Cu relative to the metal precursor, corresponding to 0.09 μmol of Cu) and 3.3 mmol base. Then 28 mmol MF (1.7 mL) and 56 mmol DI H_2_O (1.0 mL) were injected under the argon atmosphere. The reaction mixture was stirred at room temperature under 390 nm Kessil light for 20 h. Upon completion of the reaction, the pressure was carefully released at room temperature (25°C). The gas was collected in a manual burette to measure the volume (**Figure S11**), and its composition was analyzed by GC. All experiments were performed at least twice, the average gas pressures and values are shown with standard deviations <5 %.


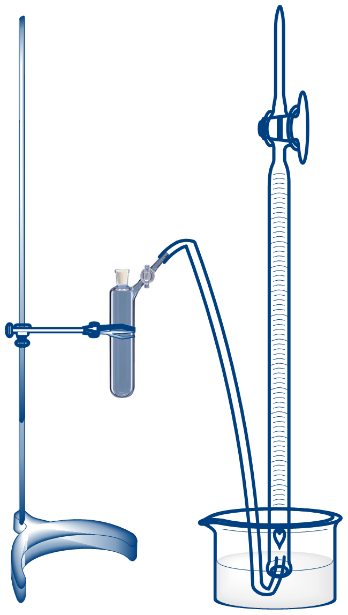


Figure S11. Illustration of the semi-open burette system for photocatalytic MF dehydrogenation.

## 4.2 Calculation of gas volume and catalytic performance

$V_{mH_{2,}25 {}^{o}C}$and $V_{m{CO}_{2}, 25 {}^{o}C}$, are the molar volumes of H_2_ and CO_2_ respectively calculated with the viral expansion of the Van der Waals equation.^[5]^

***Calculation of H_2_ molar volume (******0.02448 m^3^/mol in this study):***

$$V_{m_{H_{2}}}=\frac{R\cdot T}{P}+b-\frac{a}{R\cdot T}$$

Where:

- R = 8.3145 m^3^.Pa.mol^-1^.K^-1^
- T = 273.15 + room temperature (°C) K
- P = 101325 Pa
- a = 24.7 × 10^-3^ Pa.m^6^.mol^-2^
- b = 26.6 × 10^-6^ m^3^.mol^-1^

***Calculation of CO_2_ molar volume (0.02436 m^3^/mol in this study):***

$$V_{m_{{CO}_{2}}}=\frac{R\cdot T}{P}+b-\frac{a}{R\cdot T}$$

Where:

- R = 8.3145 m^3^.Pa.mol^-1^. K^-1^
- T = 273.15 + room temperature (°C) K
- P = 101325 Pa
- a = 36.5 × 10^-2^ Pa.m^6^.mol^-2^
- b = 42.7 × 10^-6^ m^3^.mol^-1^

***Calculation of CO molar volume (0.02518 m^3^/mol in this study):***

$$V_{m_{CO}}=\frac{R\cdot T}{P}+b-\frac{a}{R\cdot T}$$

Where:

- R = 8.3145 m^3^.Pa.mol^-1^. K^-1^
- T = 273.15 + room temperature (°C) K
- P = 101325 Pa
- a = 15.7 × 10^-2^ Pa.m^6^.mol^-2^
- b = 39.1 × 10^-6^ m^3^.mol^-1^

***Gas generation performance of catalysts (P_cat_, mmol/g_cat_):***

$$P_{cat} = \frac{n_{\mathrm{gas}}}{m_{cat}} = \frac{\frac{C{\cdot V}_{gas}}{V_{m}}}{m_{cat}}$$

Where:

- **P_cat_**: the gas generation performance of the catalyst (mmol/g_cat_)
- n_gas_: the amount of gas generated (mmol)
- m_cat_: the mass of the catalyst used (g)
- C: the concentration of the gas determined by GC, expressed in ppm (v/v)
- V_gas_: the tolal volume of gas
- V_m_: standard molar volume of gas

## 4.3 Optimization of reaction conditions

Table S2. Effect of different catalysts

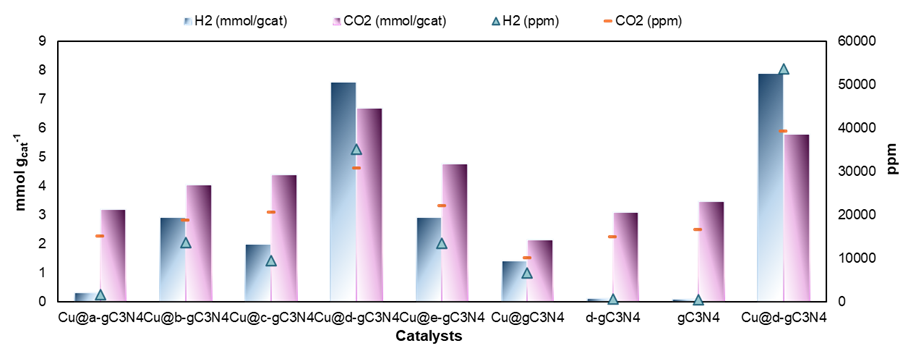

[a] Cat 20 mg, 0.18 μmol Cu

Table S3. Screening of different bases

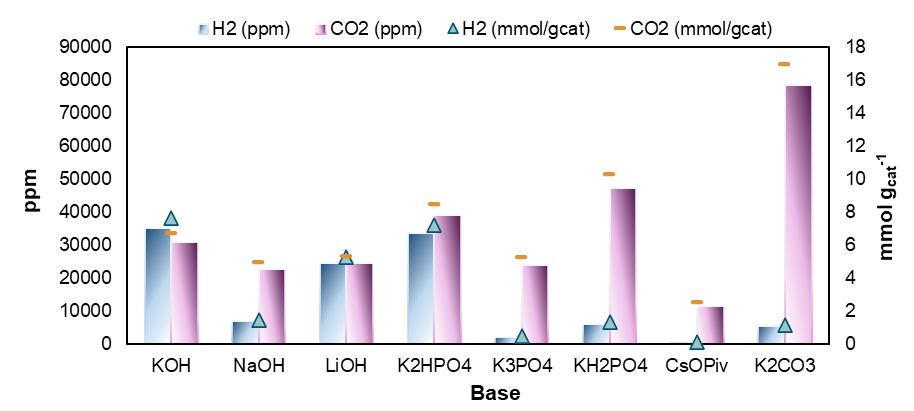

Table S4. Screening of different solve**nts**

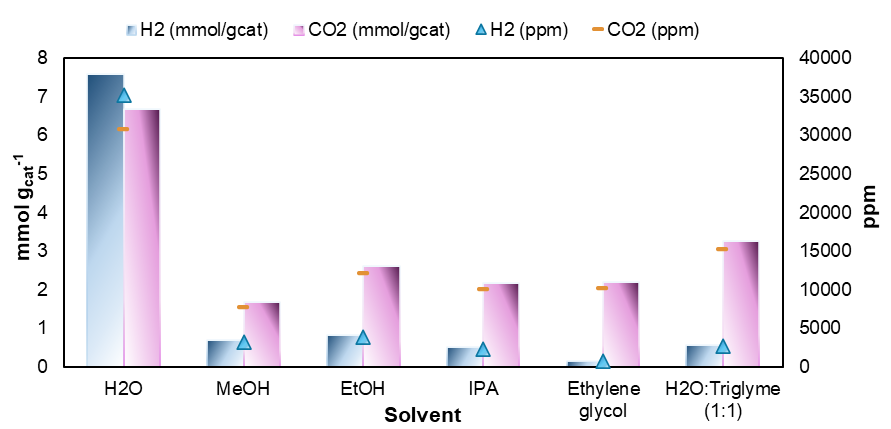

Table S5. Screening of base amount

**Table S6. Screening of H_2_O amounts**

Table S7. Other control experiments on MF dehydrogenation

## **4.4** Apparent quantum yield (AQY) measurement

The amount of formed H_2_ (mol):

$$n_{H_{2}} = \frac{C\cdot V_{gas}}{V_{m}}= \frac{35166 ppm\cdot{10}^{-6}\cdot52.9\cdot{10}^{-6}m^{3}}{0.02448 m^{3}/mol}=7.6 x {10}^{-5}\mathrm{mol}$$

The light intensity of blue LED strip light (λ = 390 nm, 40W) was 0.10 W/cm^2^.

$$\eta=\frac{N_{e}}{N_{p}}\times100\%=\frac{2\times n\times N_{A}}{\frac{S\times P\times t}{h\times\frac{c}{\lambda}}}\times100\%=\frac{2\times n_{H_{2}}\times N_{A}\times h\times c}{S\times P\times t\times\lambda}\times100\%=\frac{2*7.6\times{10}^{-5}*6.022\times{10}^{23}\times6.63\times{10}^{-34}\times3\times{10}^{8}}{2 \pi\times0.10\times20\times3600\times390\times{10}^{-9}}\times100\%=0.103 \%$$

Where, **M** represents the amount of formed product (mol), **N_A_** is the Avogadro constant (6.022 x 10^23^ mol^-1^ ), **h** is the Planck constant (6.63 x 10^-34^ J·s), **c** is the light speed (3 x 10^8^ m·s^-1^), **S** is the irradiation area (2 x π cm^2^), **P** is the light intensity (0.10 W·cm^-2^), **t** is irradiation time (20 x 3600 s), **λ** is the wavelength of light (m).

## **4.5** **Comparison of dehydrogenation in different substance**s

**Table S8.** **Dehydrogenation of H_2_O, MeOH, FA, HCOOK and MF**

Reaction conditions: an oven dried 25 mL Schlenk tube containing a magnetic stir bar was charged with 10 mg Cu@d-gC_3_N_4_ (0.2 wt% Cu relative to the metal precursor, corresponding to 0.09 μmol of Cu) and 3.3 mmol KOH (185 mg). Then 28 mmol substrate and 56 mmol DI H_2_O (1.0 mL) were injected under the argon atmosphere. The reaction was performed under 390 nm Kessil light for 20 h. Upon completion of the reaction, the pressure was carefully released at room temperature (25 °C). The gas was collected in a manual burette to measure the volume, and its composition was analyzed by GC. All experiments were performed at least twice, the average gas pressures and values are shown with standard deviations <5 %. [a] The reaction was conducted with 1 mL DI H_2_O. [b] Use 15 mL Schlenk tube.

## 4.6 Time course experiment

Table S9. Time course experiments of dehydrogenation

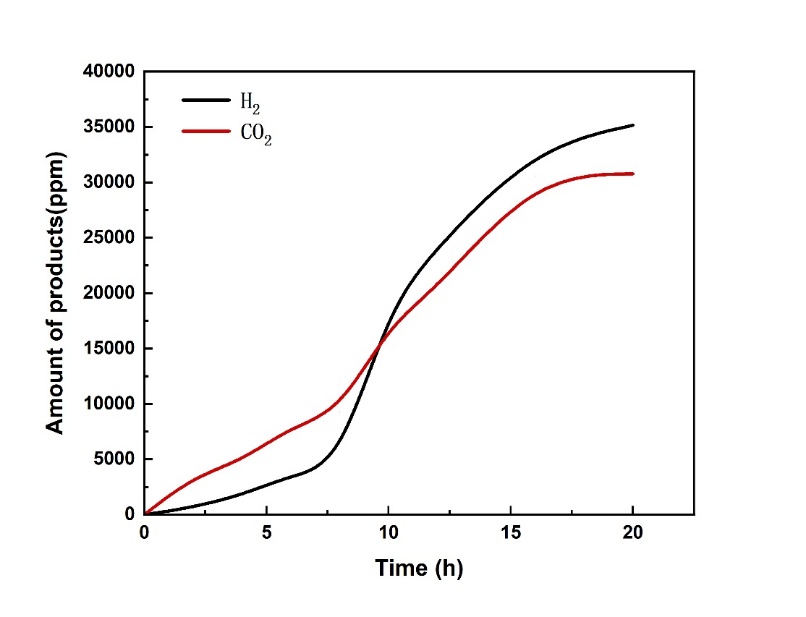

Reaction conditions: an oven dried 25 mL Schlenk tube containing a magnetic stir bar was charged with 10 mg Cu@d-gC_3_N_4_ (0.2 wt% Cu relative to the metal precursor, corresponding to 0.09 μmol of Cu) and 3.3 mmol KOH (185 mg). Then 28 mmol MF and 56 mmol DI H_2_O (1.0 mL) were injected under the atmosphere of the argon. The reaction was performed under 390 nm Kessil light for **xx h**. Upon completion of the reaction, the pressure was carefully released at room temperature (25 °C). The gas was collected in a manual burette to measure the volume, and its composition was analyzed by GC. All experiments were performed at least twice, the average gas pressures and values are shown with standard deviations <5 %.

## 4.7 Conversion of MF dehydrogenation reaction

Table S10. Conversion of MF

Reaction conditions: an oven dried 25 mL Schlenk tube containing a magnetic stir bar was charged with 5 mg Cu@d-gC_3_N_4_ (0.2 wt% Cu relative to the metal precursor, corresponding to 0.045 μmol of Cu) and KOH (3-4 mg). Then 0.5 mmol MF and DI H_2_O (0.5 mL) were injected under the argon atmosphere. The reaction was performed under 390 nm Kessil light for 20 h. Upon completion of the reaction, the pressure was carefully released at room temperature (25 °C). Conversion and yields were determined by ^1^H NMR analysis of the reaction mixture, using mesitylene as the internal standard and acetone-d_6_ as the NMR solvent.

## 4.8 Long-term reaction experiments

Table S11. Hydrogen production over time ^[a][b][c]^

Reaction conditions: [a] an oven dried 25 mL Schlenk tube containing a magnetic stir bar was charged with 10 mg Cu@d-gC_3_N_4_ (0.2 wt% Cu relative to the metal precursor, corresponding to 0.09 μmol of Cu) and 3.3 mmol KOH (185 mg). Then 28 mmol MF and DI H_2_O (1 mL) were injected under the argon atmosphere. The reaction was performed under 390 nm Kessil light for 1 day. Upon completion of the reaction, the pressure was carefully released at room temperature (25 °C). The gas was collected in a manual burette to measure the volume, and its composition was analyzed by GC. [b] After gas detection, the 25 mL Schlenk tube with reaction mixture was cooled with liquid nitrogen, and the gas was removed using a vacuum pump. The mixture was then thawed and purged with argon gas. This freeze-pump-thaw cycle procedure was repeated 3 times, and the reaction was continued under 390 nm light for an additional 1 day. [c] The procedure outlined in [b] was repeated after each time, continuing until the final day of the reaction.

## 4.9 Sunlight-driven dehydrogenation of MF

Table S12. Sunlight-driven hydrogen production

Figure S12. Sunlight and darkness duration.

Reaction conditions: an oven dried 25 mL Schlenk tube containing a magnetic stir bar was charged with 10 mg Cu@d-gC_3_N_4_ (0.2 wt% Cu relative to the metal precursor, corresponding to 0.09 μmol of Cu) and 3.3 mmol KOH (185 mg). Subsequently, 28 mmol MF and DI H_2_O (1 mL) were injected under the argon atmosphere. The reaction mixture was stirred under the sunlight. Upon completion of the reaction, the pressure was carefully released at room temperature (25 °C). The gas was collected in a manual burette to measure the volume, and its composition was analyzed by GC. As shown in **Figure S12**, the 20 h of sunlight was accumulated over a period of 6 days, with the remaining time under dark conditions.

## 4.10 Scale-up reaction of MF dehydrogenation

Table S13. Scale-up reaction

Reaction conditions: an oven dried 120 mL Schlenk tube containing a magnetic stir bar was charged with 100 mg Cu@d-gC_3_N_4_ (0.2 wt% Cu relative to the metal precursor, corresponding to 0.9 μmol of Cu) and 33 mmol KOH (1.85 g). Subsequently, 200 mmol MF and DI H_2_O (7.5 mL) were injected under the argon atmosphere. The reaction mixture was stirred under 390 nm Kessil light for 24 h. Upon completion of the reaction, the pressure was carefully released at room temperature (25 °C). The gas was collected in a manual burette to measure the volume, and its composition was analyzed by GC.

## 4.11 Light on-off experiments

An oven dried 25 mL Schlenk tube containing a magnetic stir bar was charged with 10 mg Cu@d-gC_3_N_4_ (0.2 wt% Cu relative to the metal precursor, corresponding to 0.09 μmol of Cu) and 3.3 mmol KOH (185 mg). Subsequently, 28 mmol MF and DI H_2_O (1 mL) were injected under the argon atmosphere. The reaction mixture was stirred at room temperature under 390 nm Kessil light, and the light being turned on and off every 2 hours. After each reaction was completed, the pressure was carefully released at room temperature (25°C). The gas was collected in a manual burette to measure the volume, and its composition was analyzed by GC. After each reaction period, the 25 mL Schlenk tube containing reaction mixture was cooled with liquid nitrogen, and the gas was removed using a vacuum pump. The mixture was then thawed and purged with argon gas. This freeze-pump-thaw cycle was repeated 3 times, and the reaction was continued under 390 nm Kessil light for an additional 2 hours. As shown in **Figure S13**, the reaction conducted without Kessil light (390 nm) exhibited an extremely slow reaction rate, emphasizing the crucial role of light irradiation.

Figure S13. Light on-off experiments.

## 4.12 Catalyst recycling

Figure S14. Catalyst recycling.

Reaction conditions: [a] an oven dried 120 mL Schlenk tube containing a magnetic stir bar was charged with 10 mg Cu@d-gC_3_N_4_ (0.2 wt% Cu relative to the metal precursor, corresponding to 0.09 μmol of Cu) and 3.3 mmol KOH (185 mg). Subsequently, 28 mmol MF and DI H_2_O (1 mL) were injected under the argon atmosphere. The reaction mixture was stirred at room temperature under 390 nm Kessil light for 24 h. Upon completion of the reaction, the pressure was carefully released at room temperature (25°C). The gas was collected in a manual burette to measure the volume, and its composition was analyzed by GC. [b] The catalyst was separated and collected from the reaction mixture. The solid catalyst was washed 5 times with water, acetone, and CH_2_Cl_2_ separately. Then, the solid catalyst was separated from the liquid using a centrifuge and the solvent was removed under vacuum to obtain the dry catalyst. Finally, the catalyst was then reused in the next cycle according to step [a].

## 4.13 Quenching experiments

An oven dried 10 mL Schlenk tube containing a magnetic stir bar was charged with Cu@d-gC_3_N_4_, KOH and quencher (DMPO or TEMPO). Then MF and DI H_2_O were injected under the argon atmosphere. The reaction was performed under 390 nm Kessil light for 20 h. Upon completion of the reaction, the pressure was carefully released at room temperature (25 °C). The gas was collected in a manual burette to measure the volume, and its composition was analyzed by GC. The mixture was filtered rapidly, and the filtrate was analyzed by HRMS. **Figure S15** shows that addition of either 2,2,6,6‑tetramethyl‑1‑piperidinyloxyl (TEMPO) or 5,5‑dimethyl‑1‑pyrroline N‑oxide (DMPO) to the reaction mixture completely inhibits the reaction, providing clear evidence for a radical‑mediated pathway.

Figure S15. Radical-trapping experiments and HRMS analysis of trapped by DMPO and TEMPO.

## 4.14 Reaction rate determination

To provide further information about the reaction mechanism, in the initial stage of the reaction, the reaction rate was measured using different concentrations of KOH and H_2_O respectively, the results as shown in **Figure S16**.

Figure S16. Reaction rate determination w.r.t. reactants involved.

## 4.15 Electron paramagnetic resonance (EPR) studies

***Experiments***

In-situ EPR analysis of the reaction under three different conditions was performed. In the first experiment, the reaction mixture, consisting of the catalyst (Cu@d-gC_3_N_4_), MF, H₂O, and KOH, was placed in a round-bottom flask and sealed with a septum. To maintain an inert atmosphere, Ar was bubbled through the mixture. DMPO was then added as a spin trap, and a 50 µL aliquot was transferred into a Hirschmann glass microcapillary tube for EPR analysis. Spectra were recorded immediately at RT. In the second and third experiments, the same procedure was followed. In one experiment, d_1_-MF was mixed with H₂O and KOH, while in the other, d_1_-MF was mixed with D_2_O and KOD instead of H_2_O and KOH. The reaction mixtures in all three experiments were prepared with reactant ratios similar to those used in the catalytic tests. Each experiment was measured both in the dark and after irradiation with 390 nm light. In situ EPR spectra of TEMPO spin adducts measured at 293 K for mixtures containing the Cu@d-gC_3_N_4_ catalyst with MF, H_2_O, and KOH under irradiation with 390 nm light.

***Analysis***

The reaction was also investigated using in situ EPR with DMPO as a spin trap to detect short-lived radicals. In experiments involving MF, H_2_O, and KOH, complex spectra were observed in the dark **(Figure S17A)**, indicating the formation of three DMPO-adducts corresponding to two carbon-centered radicals and •OH (a_N_ = a_H_ = 14.4 G) radicals. One of the DMPO-adduct signals, with hyperfine splitting of a_N_ = 15.3 G and a_H_ = 18.3 G, is attributed to the DMPO/•COOMe spin adduct, while another, with hyperfine splitting of a_N_ = 15.4 G and a_H_ = 22.02 G, is characteristic of the DMPO/•COO adduct. Upon irradiation, the DMPO/•COOMe spin adduct and •OH rapidly disappeared, while the EPR signals for the DMPO/•COO adduct became dominant over time. In contrast, experiments using d_1_-MF, whether with H_2_O and KOH or D_2_O and KOD, showed no signal in the dark **(Figure S17B)**. Upon irradiation, a six-line signal characteristic of only the DMPO/•COO spin adduct appeared. The absence of •OH and the DMPO/•COOMe adduct observed with MF **(Figure S17A)** can be attributed to their slow formation and quick transformation of •COOMe to •COO (it could be also due to other reasons). When TEMPO was added to a solution containing Cu@d-gC_3_N_4_, MF, H_2_O, and KOH, the three-line EPR signal characteristic of the *N*-center radical of TEMPO decreased over time, likely due to the formation of TEMPO-OH **(Figure S17C)**, as evidenced by HR-MS.

Figure S17. In situ EPR spectra of DMPO spin adducts measured at 293 K for mixtures containing the Cu@d-gC_3_N_4_ catalyst with (A) MF, H_2_O, and KOH; and (B) d_1_-MF, H_2_O, and KOH. Similar spectra to (B) were observed when Cu@d-gC_3_N_4_ and d_1_-MF were mixed with D_2_O and KOD instead of H_2_O and KOH. (C) In situ EPR spectra of DMPO spin adducts measured at 293 K for mixtures containing the Cu@d-gC_3_N_4_ catalyst with MF, H_2_O, and KOH under irradiation with 390 nm light.

## 4.16 NMR spectra of the product and reaction intermediates

Reaction conditions: an oven dried 25 mL Schlenk tube containing a magnetic stir bar was charged with 5 mg Cu@d-gC_3_N_4_ (0.2 wt% Cu relative to the metal precursor, corresponding to 0.045 μmol of Cu) and KOH (3-4 mg). Then 0.5 mmol MF and DI H_2_O (0.5 mL) were injected under the argon atmosphere. The reaction was performed under 390 nm Kessil light for 20 h. Upon completion of the reaction, the pressure was carefully released at room temperature (25 °C). Conversion and yields were determined by ^1^H NMR analysis of the reaction mixture, using mesitylene as the internal standard and acetone-d_6_ as the NMR solvent. Acetone-d_6_ is a polar solvent miscible with both water and methyl formate, commonly used as an NMR solvent or co-solvent. Mesitylene is a nonpolar aromatic hydrocarbon. After choosing acetone-d_6_ as the deuterated solvent, the reaction mixture forms a fully homogeneous solution due to acetone’s ability to dissolve hydrophobic compounds and the addition of only a small amount of mesitylene.


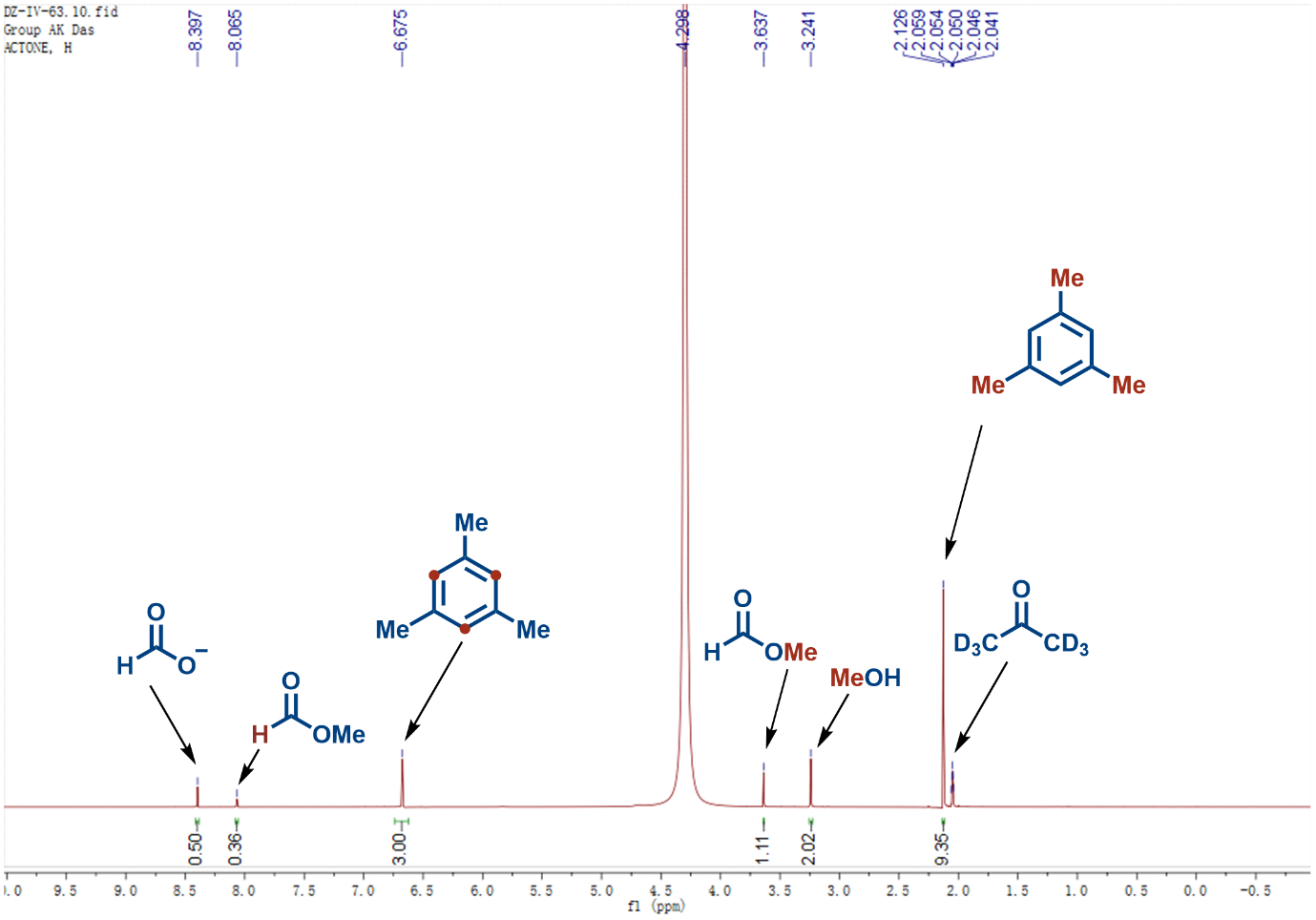


## 4.17 Gas Chromatogram of the collected reaction gas mixture


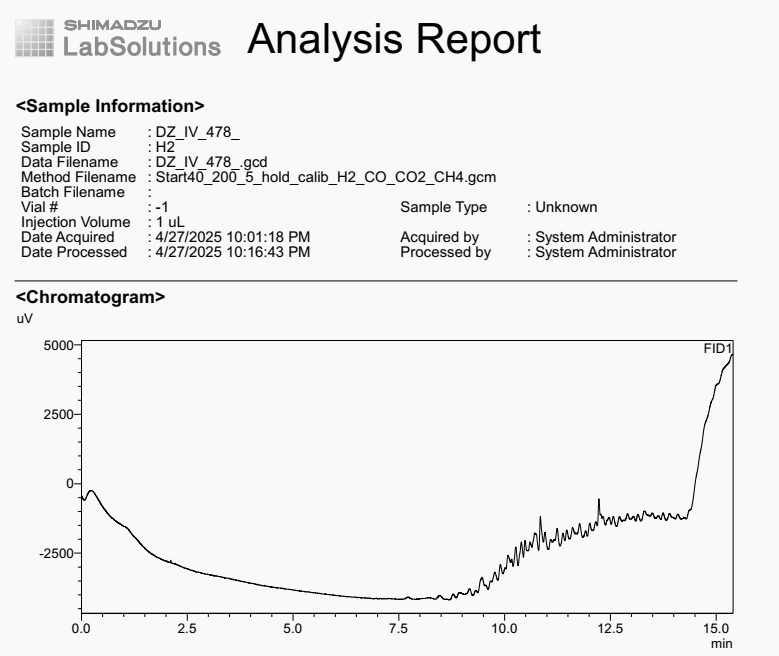


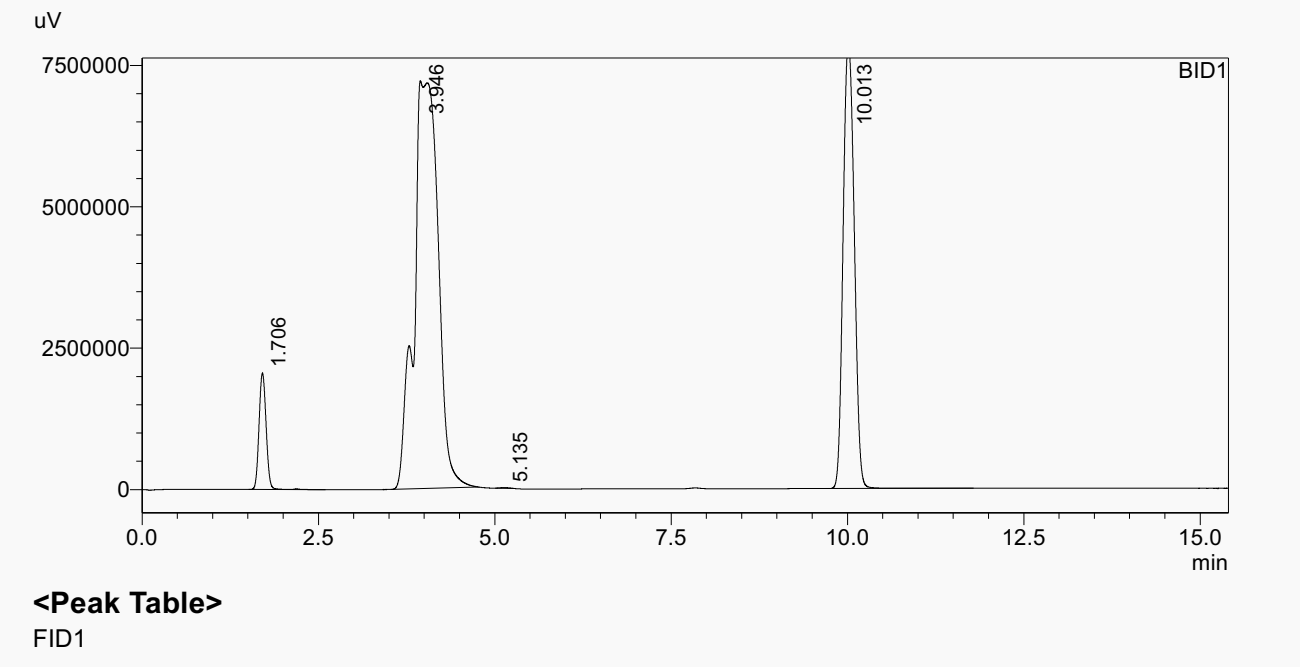

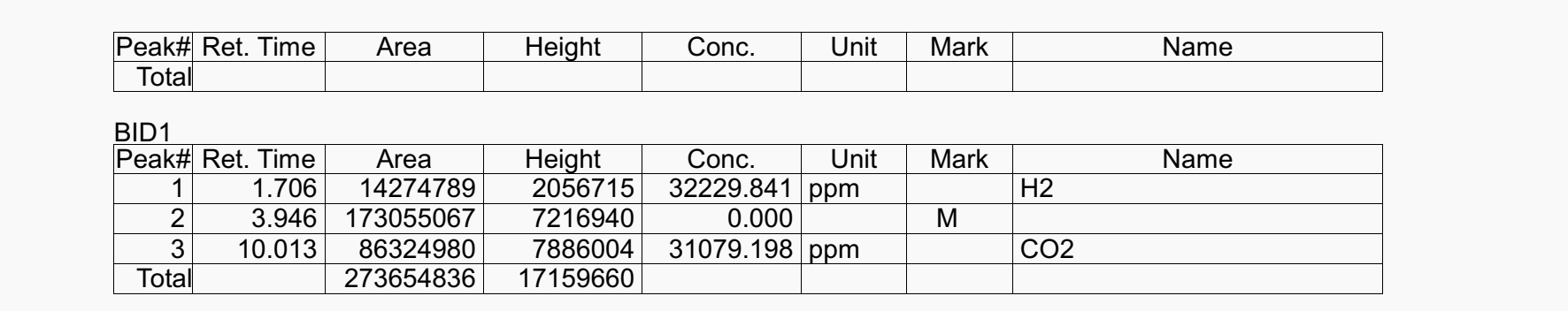


CO

CO_2_

H_2_

He and Ar


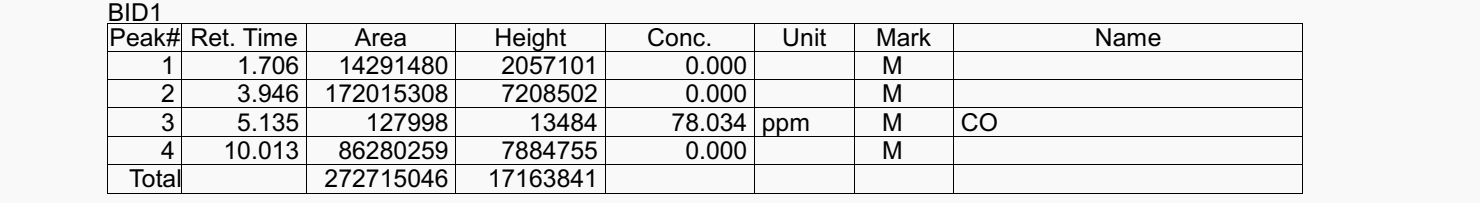
Figure S18. Typical GC spectra using He carrier gas.

## 4.18 Intermediate detection in aqueous reforming of methyl formate

Reaction conditions: an oven dried 10 mL Schlenk tube containing a magnetic stir bar was charged with Cu@d-gC_3_N_4_, KOD and TEMPO (2.0 equiv, 1 g). Then methyl formate-d1 and D_2_O were injected under the argon atmosphere. The reaction was performed under 390 nm Kessil light for 20 h. Upon completion of the reaction, the pressure was carefully released at room temperature (25 °C). The mixture was filtered rapidly, and the filtrate was analyzed by HRMS. The result is shown in **Figure S19**.


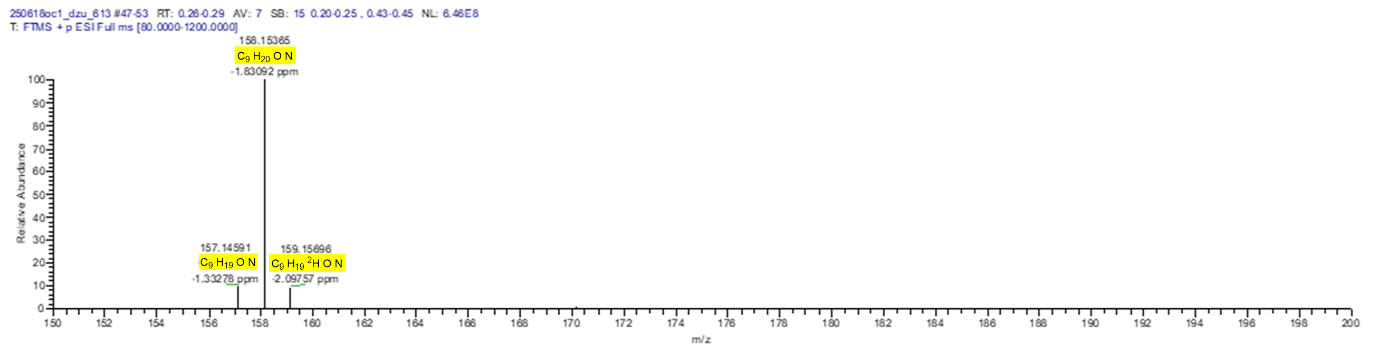


Figure S19. HRMS analysis of TEMPO-trapped intermediates.

Reaction conditions: an oven dried 10 mL Schlenk tube containing a magnetic stir bar was charged with Cu@d-gC_3_N_4_ and KOD. Then methyl formate-d1 and D_2_O were injected under the argon atmosphere. The reaction was performed under 390 nm Kessil light for 20 h. Upon completion of the reaction, the pressure was carefully released at room temperature (25 °C). The mixture was filtered rapidly, and the filtrate was analyzed by NMR. The result is shown below.

# 5. DFT Calculations

## 5.1 Computational methodology

All structures have been optimized at first at the M06L^[6]^ level of density functional theory in combination with the TZVP basis set^[7]^ in gas phase (M06L/TZVP/FOpt). All optimized structures were further characterized either as energy minimums without imaginary frequencies or transition states with only one imaginary frequency by frequency calculations at the same level of theory. Based on the M06L/TZVP optimized geometries in gas phase, M06L/TZVP single-point energy calculations and thermal correction to Gibbs free energies at 298 K under the consideration of solvation effect based on solute electron density (SMD) model for water as solvent using self-consistent reaction field theory^[8]^ were carried out (M06L-SCRF/TZVP//M06L/TZVP/SP). Therefore, the corrected Gibbs free energy (ΔG) at 298 K were used for our energetic discussion and comparison. All calculations were carried out by using the Gaussian 16 program.^[9]^

## 5.2 Benchmark testing

Based on our experimental studies and to illustrate the reaction mechanisms, we further carried out detailed DFT computation (M06L-SCRF/TZVP), and this methodology has been successfully used for the photoredox-nickel cooperatively catalyzed radical allylic silylation of allyl acetates.^[10]^ At first the reaction thermodynamics of the stoichiometric hydrolysis of methyl formate [HCOOCH_3_ + H_2_O = HCOOH + CH_3_OH] was computed. For gas phase reaction under the standard condition, the computed reaction is endothermic by 3.71 kcal/mol, in agreement with the value [Δ_f_*H*°(g) = 4.66 kcal/mol] deduced from the standard enthalpy of formation (Δ_f_*H*°_g_). The reaction is endergonic in gas phase (Δ_f_*G*°(g) = 2.94 kcal/mol). In water solution, the computed reaction is endothermic by 4.88 kcal/mol, stronger than that (Δ_f_*H*°(l) = 1.65 kcal/mol) deduced from the standard enthalpy of formation, and this deviation can come from difference between molecules in their condensed forms and the solvated states in water solution. The reaction is endergonic in water solution (Δ_f_*G*°(l) = 3.81 kcal/mol). Nevertheless, all these show that the hydrolysis of methyl formate is not favorable thermodynamically, and only a larger amount of water can shift the reaction towards the formation of formic acid and methanol.

## 5.3 DFT study on the dehydrogenation of MF

### 5.3.1 Reaction of methyl formate

Firstly, we computed the reaction between OH radical and methyl formate, and there are three possible routes (**Scheme S1**). It is found that the abstraction of the formyl C-H bond has the lowest Gibbs free energy barrier (TS1, 2.28 kcal/mol) and is exergonic (-16.34 kcal/mol), and the abstraction of the methyl C-H bond has higher Gibbs free energy barrier (TS2, 5.02 kcal/mol) and is exergonic (-16.13 kcal/mol). The substitution route has a stepwise process along with the attack of the OH radical (TS3, 17.35 kcal/mol) and the formation of a radical intermediate (9.87 kcal/mol) and the release of the methoxy radical (TS4, 11.49 kcal/mol) resulting in the formation of formic acid, and this route is also exergonic (-14.90 kcal/mol). The most kinetically favored route is the abstraction of the formyl C-H bond leading to the formation methoxy carbonyl radical (CH_3_O-CO).

Scheme S1. Reaction routes between OH radical and methyl formate

### 5.3.2 Reaction of methoxy carbonyl radical

Based on the formed methoxy carbonyl radical as the first intermediate, we computed the stability towards dissociation into CO and methoxy radical as well the subsequent reaction with OH radical (**Scheme S2**). It is found that the dissociation of methoxy carbonyl radical into CO and methoxy radical has a very high Gibbs free energy barrier (**TS5**, 19.54 kcal/mol) and is endergonic (8.25 kcal/mol), and this step is neither kinetically nor thermodynamically favored. Alternatively, the recombination of OH radical and methoxy carbonyl radical forming methoxy formic acid is highly exergonic (-96.64 kcal/mol). In basic solution, methoxy formic acid can be deprotonated to methoxy formate, which can be photochemically oxidized to methoxy formate radical. The subsequent dissociation of methoxy formate radical into CO_2_ and methoxy radical has a low Gibbs free energy barrier (**TS6**, 7.97 kcal/mol) and is exergonic (-18.92 kcal/mol).

At this step, one can see that the first reaction is the formal methyl formate hydrolysis with the formation of H_2_, CO_2_ and methanol [HCOOCH_3_ + H_2_O = H_2_ + CO_2_ + CH_3_OH]. The next step should be the reaction of the formed methoxy radical.

Scheme S2. Stability and reaction of methoxy carbonyl radical

### 5.3.3 Reaction of methoxy radical

There are several reaction possibilities for methoxy radical (**Scheme S3**). It is found that methoxy radical reaction with H_2_O resulting in methanol and OH radical is endergonic (18.71 kcal/mol) and methoxy radical dissociation into formaldehyde and hydrogen atom is also endergonic (20.60 kcal/mol), and both reactions are not favored thermodynamically. On the contrary, the reaction of methoxy radical and OH radical into formaldehyde and H_2_O is highly exergonic (-86.76 kcal/mol) and very favored thermodynamically. It is also possible that methoxy radical can react with H atom to form methanol, which can be deprotonated to methanolate in basic solution, and methanolate can be photochemically oxidized to methoxy radical for further reactions.

Scheme S3. Stability and reaction of methoxy radical

### 5.3.4 Reaction of formaldehyde

It is known experimentally that formaldehyde can react with water to form methanediol, and this reaction is computed to be slightly endergonic (4.30 kcal/mol), and excess water can shift the reaction towards the formation of methanediol (**Scheme S4**). In the basic solution, methanediol can be further deprotonated by base to hydroxyl methanolate, which can be photochemically oxidized to the corresponding hydroxyl methoxyl radical. Further reaction of hydroxyl methoxyl radical reacts with OH radical leading to formic acid, and this reaction is highly exergonic (-106.94 kcal/mol).

Scheme S4. Reaction for formaldehyde to formic acid

### 5.3.5 Reaction of formic acid

Under basic conditions, formic acid exits in the form of formate, which can be photochemically oxidized to corresponding radical (**Scheme S5**, 138.45 kcal/mol). It is found that the formed radical can either dissociates thermodynamically favorable to CO_2_ and hydrogen atom (-12.38 kcal/mol) or reacts with OH radical to CO_2_ and H_2_O, and the last step is very favorable thermodynamically (-120.25 kcal/mol).

Scheme S5. Reaction for formic acid

### 5.3.6 Proposed reaction mechanism

The whole reaction can be divided into two sequences (**Scheme S6**), and the first one is the conversion of methyl formate to CO_2_ and methoxy radical, undergoing two transition states and one photo-oxidation step; and the second sequence is the conversion of methoxy radical to CO_2_, undergoing two photo-oxidation steps.


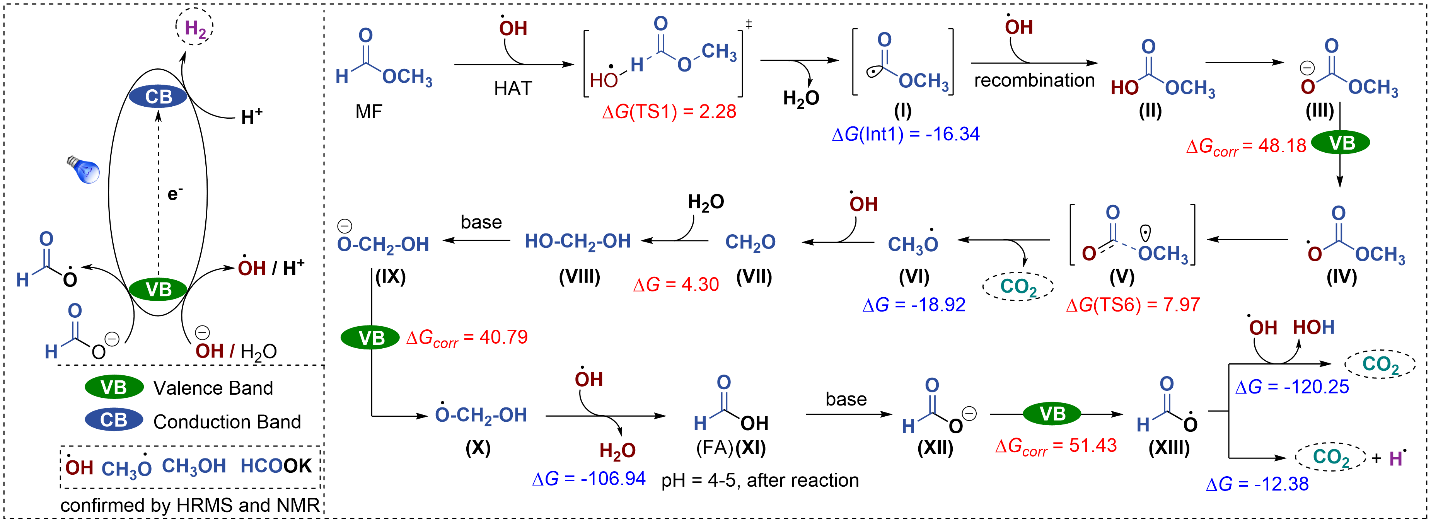


Scheme S6. Proposed reaction mechanism

### 5.3.7 Gibbs free energy correction for radical conversion reactions

In the mechanism discussed above, several reactions involving anion-to-radical conversion are involved. When calculating the free energy for such reactions, the energy of the released electron is assumed to be zero. In reality, however, this leads to consistently overestimated Gibbs free energy changes. To get more accurate energy in the conversion reaction of R^−^ − e^−^ → R•, we hereby assume the equilibrium of the reaction H^+^(aq) + R^-^ ↔ HR. For example, the reaction H^+^(aq) + OH^−^ (aq) ↔ H_2_O(aq) is assumed to be equilibrium. It could be also expressed as (H^+^(aq) + e^–^) + (OH^−^(aq) − e^–^) ↔ H_2_O(aq). It is generally assumed that in photocatalysis or electrocatalysis the single-electron transfer reaction (H^+^ + e^-^ = 1/2 H_2_) occurring at the standard hydrogen electrode has zero Gibbs free energy, i.e., Δ*G*(H^+^) + e^-^ = 1/2Δ*G*(H_2_). Therefore, the free energy of OH^−^(aq) − e^–^ can be determined as:

Δ*G*(OH^−^(aq) − e^–^) = *G*(H_2_O(aq)) − *G*(H^+^(aq) + e^–^) = *G*(H_2_O (aq)) -1/2 *G*(H_2_).

Then, for the OH^−^ − e^−^ → •OH conversion step, Δ*G* = *G*(•OH)(aq) - *G*(H_2_O (aq)) +1/2*G*(H_2_).

The free energy change for other radical conversion reactions can be obtained in the same way. Based on this rationale, the Gibbs free energy changes for reactions (**III**, -O-CO-OCH_3_) → (**IV**, •O-CO-OCH_3_), (**IX**, ^−^OCH_2_OH) → (**X**, •OCH_2_OH), (**XII**, HCOO^-^) → (**XIII**, HCOO•) and OH^−^→ •OH in the aforementioned mechanistic scheme are corrected to 48.18, 40.79, 51.43 and 61.31 kcal mol⁻¹, respectively, and OH- seems to be the most difficult one to be oxidized.

All these values are lower than that of the Kessil lamps (390 nm = 73.79 kcal/mol).

Table S14. M06L/TZVP computed total electronic energies (HF, au), zero-point vibrational energies (ZPE, au), sum of electronic and thermal enthalpies (Htot, au), sum of electronic and thermal free energies (Gtot, au), number of Imaginary frequencies (NImag) in gas phase, as well as M06L-SCRF/TZVP//M06L/TZVP/SP computed sum of electronic and thermal Enthalpies (Htot, au) and sum of electronic and thermal free energies (Gtot, au) in solution.

|  | M06L/TZVP/FOpt | M06L-SCRF/TZVP/ |
| --- | --- | --- |
|  | HF=-76.4414148  ZPE=0.021401  NImag=0  Htot=-76.416235  Gtot= -76.437657 | Htot= -76.429331  Gtot= -76.450753 |
| HO^-^ Anion | HF=-75.7864373  ZPE=0.008443  NImag=0  Htot=-75.774690  Gtot= -75.794251 | Htot= -75.937456  Gtot= -75.957018 |
|   HO-Radical | HF=-75.7505541  ZPE=0.008447  NImag=0  Htot=-75.738803  Gtot= -75.759030 | Htot= -75.747628  Gtot= -75.767856 |
|   Methyl formate | HF=-229.1148066  ZPE=0.061733  NImag=0  Htot=-229.047547  Gtot= -229.080057 | Htot= -229.050714  Gtot= -229.082868 |
|   **TS1** | HF=-304.8728054  ZPE= 0.067788  NImag=1 (-893.0759)  Htot= -304.797236  Gtot= -304.836439 | Htot= -304.808038  Gtot= -304.847089 |
|   **Intermediate 1** | HF=-228.4486447  ZPE=0.049010  NImag=0  Htot=-228.394123  Gtot=-228.427183 | Htot= -228.393155  Gtot= -228.426004 |
|   **TS2** | HF=-304.8683098  ZPE=0.068057  NImag=1 (-1848)  Htot=-304.793177  Gtot= -304.830410 | Htot= -304.804231  Gtot= -304.842730 |
|   **Intermediate 2** | HF=-228.445111  ZPE=0.046267  NImag=0  Htot=-228.392793  Gtot= -228.427936 | Htot= -228.393501  Gtot= -228.425677 |
|   **TS3** | HF=-304.8583852  ZPE= 0.073885  NImag=1 (-502.0163)  Htot= -304.777705  Gtot= -304.814067 | Htot= -304.786437  Gtot= -304.823077 |
|   **Intermediate 3** | HF=-304.8700128  ZPE=0.075929  NImag=0  Htot=-304.787225  Gtot= -304.823731 | Htot= -304.797805  Gtot= -304.834988 |
|   **TS4** | HF=-304.8651998  ZPE=0.074137  NImag=1 (-522.4228)  Htot=-304.784386  Gtot= -304.820697 | Htot= -304.795934  Gtot= -304.832406 |
| HCOOH  Formic acid | HF=-189.807029  ZPE= 0.033753  NImag=0  Htot= -189.769172  Gtot= -189.797345 | Htot= -189.777283  Gtot= -189.805471 |
|   Methoxy radical | HF=-115.0768932  ZPE= 0.034627  NImag=0  Htot= -115.038402  Gtot= -115.065152 | Htot= -115.042225  Gtot= -115.068991 |
|   **TS5** | HF=-228.4145129  ZPE=  NImag=1 (-264.7031)  Htot= -228.363737 Gtot= -228.398504 | Htot= -228.359242  Gtot= -228.394872 |
| CO  Carbon Monoxide | HF=-113.3383397  ZPE=0.005029  NImag=0  Htot=-113.330005  Gtot= -113.352435 | Htot= -113.321440  Gtot= -113.343870 |
| CH3OH  Methanol | HF=-115.7442577  ZPE=0.051299  NImag=0  Htot=-115.688697  Gtot= -115.715688 | Htot= -115.694977  Gtot= -115.722070 |
|   methoxy formic acid | HF=-304.3774268  ZPE= 0.067564  NImag=0  Htot= -304.303628  Gtot= -304.338295 | Htot= -304.313454  Gtot= -304.347863 |
|   Methoxy formate | HF=-303.8201415  ZPE= 0.053107  NImag=0  Htot= -303.760904  Gtot= -303.795532 | Htot= -303.867984  Gtot= -303.901972 |
|   Methoxy carboxylic radical | HF=-303.7074607  ZPE= 0.053658  NImag=0  Htot= -303.647617  Gtot= -303.682962 | Htot= -303.650305  Gtot= -303.685779 |
|   **TS6** | HF=-303.6892021  ZPE=0.050310  NImag=1 (-571.6056)  Htot=-303.632315  Gtot= -303.668970 | Htot= -303.638432  Gtot= -303.673085 |
| CO2  Carbon dioxide | HF=-188.6420764  ZPE=0.011906  NImag=0  Htot=-188.626612  Gtot= -188.650861 | Htot= -188.622685  Gtot= -188.646945 |
| CH2O  Formaldehyde | HF=-114.5287419  ZPE=0.026438  NImag=0  Htot=-114.498491  Gtot= -114.523300 | Htot= -114.499541  Gtot= -114.524347 |
| HO-CH2-OH  Methanediol  HO-CH2-OH-M06L | HF=-190.9885729  ZPE=0.057289  NImag=0  Htot=-190.926575  Gtot= -190.955490 | Htot= -190.939151  Gtot= -190.968242 |
|   Hydroxymethanolate | HF=-190.3874648  ZPE=0.041122  NImag=0  Htot=-190.341566  Gtot= -190.371315 | Htot= -190.459436  Gtot= -190.488213 |
|   Hydroxymethoxyl radical | HF=-190.3232294  ZPE=0.040796  NImag=0  Htot=-190.277561  Gtot= -190.308196 | Htot= -190.288684  Gtot= -190.317942 |
|  | HF=-189.2425508  ZPE=0.019957  NImag=0  Htot=-189.218675  Gtot= -189.246388 | Htot= -189.331134  Gtot= -189.358846 |
|  | HF=-189.1319687  ZPE=0.019882  NImag=0  Htot=-189.108114  Gtot= -189.136597 | Htot= -189.109725  Gtot= -189.138217 |
| H2 | HF=-1.1717114  ZPE= 0.009901  NImag=0  Htot= -1.158506  Gtot= -1.173300 | Htot= -1.155808  Gtot= -1.170602 |
| H-Radical |  | Htot= -0.498808  Gtot= -0.511823 |

Table S15. M06L/TZVP optimized Cartesian Coordinates in gas phase

|  | H,0,0.000001252,1.5814508084,-0.7051282145  O,0,0.000001252,1.5702663879,0.2551632392  H,0,0.000001252,2.5007314617,0.49290157 |
| --- | --- |
| HO- Anion | O,0,0.000001252,1.5712830625,0.2498132319  H,0,0.000001252,2.5054683625,0.5001273571 |
|   HO-Radical | O,0,0.000001252,1.5684772477,0.2490614163  H,0,0.000001252,2.5082741773,0.5008791727 |
|   Methyl formate | C,0,0.0325822504,3.341691029,0.4782492512  H,0,0.1570595604,3.086233515,-0.5848380626  O,0,-0.1501284881,4.4435158566,0.9121311205  O,0,0.1197249413,2.2170970013,1.1998231702  C,0,-0.0256378604,2.4040289281,2.6139186284  H,0,0.7499455277,3.0672538625,2.9947796384  H,0,0.0669389046,1.4180768338,3.0569207023  H,0,-0.9983111776,2.836082833,2.8456254595 |
|   **TS1** | C,0,0.0098033435,0.7382673245,-0.1327268139  O,0,0.3505142662,0.7194378582,-1.2759306532  O,0,-0.2705994629,-0.2905616313,0.6539512887  C,0,-0.1408269082,-1.5871937894,0.028106465  H,0,0.8820433464,-1.7381999387,-0.3118702406  H,0,-0.8193384514,-1.6634380605,-0.8195149721  H,0,-0.4009937913,-2.3095913611,0.7936317288  H,0,-0.1399881814,1.7768516531,0.5219827218  O,0,-0.3601450616,2.6999898517,1.3957007261  H,0,-0.6141210992,2.1732958934,2.1676688494 |
|   **Intermediate 1** | C,0,0.031245403,3.3402876908,0.4960936293  O,0,-0.1468307987,4.4632921249,0.8428276126  O,0,0.1178660143,2.2340340982,1.195361448  C,0,-0.0268530381,2.3975846169,2.6381498007  H,0,0.7547103701,3.0547078989,3.0143259272  H,0,0.0689109583,1.4027046379,3.0576310432  H,0,-1.0028626446,2.8223049789,2.8643769186 |
|   **TS2** | C,0,0.0766855343,0.0098221665,0.9102920985  H,0,-0.3322961156,0.8409565359,1.4989209473  O,0,0.5109731477,0.0828935165,-0.2031840783  O,0,0.015506955,-1.1171218817,1.6506370768  C,0,0.4751099004,-2.2778633922,1.0197762033  H,0,0.0144429556,-2.4455848642,0.0473073074  H,0,0.3932476812,-3.1042779804,1.7175957137  H,0,1.7044642235,-2.1442610947,0.7635281498  O,0,2.825800406,-1.7811803257,0.3672264948  H,0,2.5535462458,-1.1074254751,-0.2758441772 |
|   **Intermediate 2** | C,0,0.067248806,3.3499515413,0.4839674095  H,0,-0.0612513229,3.086703983,-0.572491306  O,0,0.1979670337,4.4482402569,0.937450456  O,0,0.0530038138,2.1947122957,1.2090866428  C,0,0.1973627022,2.2948316631,2.5546610319  H,0,0.3234243125,3.2697842113,2.9975598042  H,0,0.1854845067,1.3521178897,3.0713094027 |
|   **TS3** | O,0,-0.3941453607,-1.1290126396,1.3067674299  C,0,0.2172877247,-2.379037101,0.9584717301  H,0,-0.1740775616,-3.1035954828,1.6649614317  H,0,1.3002237777,-2.3080991897,1.0485912932  H,0,-0.0417840777,-2.6660447134,-0.0596341952  C,0,0.0247146221,-0.0806082631,0.5975827488  H,0,-0.5454033558,0.8048197241,0.8968124242  O,0,0.5426075191,-0.1872370197,-0.5510155591  O,0,1.6493322137,0.2948071496,1.2501767872  H,0,1.9486406786,1.0527000096,0.7247365242 |
|   **Intermediate 3** | C,0,0.0340861237,1.6055010223,0.0840081316  H,0,-0.4290107296,2.3030945671,0.7901120865  O,0,-0.5751913017,0.3394260477,0.3822875227  C,0,0.127629749,-0.7972766653,-0.1020729417  H,0,0.4048634709,-0.6826692372,-1.1513007624  H,0,-0.5486053077,-1.6416754316,-0.0078835758  H,0,1.0232482788,-0.9756381767,0.4974308666  O,0,-0.2945718968,1.746697268,-1.2094730148  O,0,1.401010639,1.6574489421,0.2956892801  H,0,1.8155335743,1.2952643635,-0.5002745929 |
|   **TS4** | O,0,-0.5339487852,-1.2577997931,1.0333182666  C,0,0.321048783,-2.3205092836,0.7607401684  H,0,0.0225715332,-3.1235951816,1.4429905789  H,0,1.3716038871,-2.0808064628,0.95676152  H,0,0.1999970689,-2.6935546251,-0.2591400428  C,0,0.1726213238,0.2881618979,0.7983599949  H,0,-0.5665812223,0.8483308719,1.3786825874  O,0,0.1310578269,0.2035054402,-0.4632214292  O,0,1.3748738383,0.344043917,1.4260638353  H,0,2.0341519262,0.0909156964,0.7628951365 |
| HCOOH  Formic acid | C,0,0.0343208976,3.3268851671,0.4808176314  H,0,0.1550954423,3.1042756318,-0.5897794737  O,0,-0.1491520978,4.4136020597,0.9470495009  O,0,0.1257535073,2.1841622306,1.180206449  H,0,0.0147453466,2.4205633768,2.1149550833 |
|   Methoxy radical | O,0,-0.0125970851,1.6000074043,0.2801004763  C,0,0.0189843605,2.9181972111,0.5766779188  H,0,1.0311448831,3.3150611631,0.7475842815  H,0,-0.5056588018,3.0218740812,1.5452224173  H,0,-0.5411072959,3.5500015192,-0.129225444 |
|   **TS5** | C,0,0.4846945553,-0.0039548418,-0.3471543781  O,0,1.0354256394,-0.3258339357,0.5928921475  O,0,-0.8939465235,1.4063088045,-0.1582542827  C,0,-1.5133875013,1.7398354744,-1.3424292657  H,0,-0.8352246617,2.1767058895,-2.0843717068  H,0,-2.2390570138,2.5210217347,-1.0739475142  H,0,-2.0829611944,0.9156005744,-1.7867927999 |
| CO  Carbon Monoxide | C,0,-0.005409772,-1.120327405,0.64563331  O,0,-0.005409772,-1.120327405,1.77551325 |
| CH3OH  Methanol | H,0,0.4613521086,1.4914735371,-0.5988435464  O,0,0.0044578129,1.5726651029,0.2421921259  C,0,0.0080653009,2.9377400781,0.6014939331  H,0,1.0194033395,3.3389666987,0.7350363831  H,0,-0.5112114415,3.0188215488,1.5543612527  H,0,-0.5193817569,3.5689060861,-0.1231094667 |
|   methoxy formic acid | C,0,-0.0569728081,0.068562136,0.9455275517  O,0,0.0796188615,1.0662025138,1.8327250436  H,0,0.0973383337,1.8766416614,1.3092969628  O,0,-0.1373113402,0.1983235206,-0.2481153248  O,0,-0.0866915988,-1.0779043567,1.6239030535  C,0,-0.2299371613,-2.2359477902,0.7983139723  H,0,0.6026610016,-2.3186154603,0.1009402709  H,0,-1.1600769675,-2.1947527208,0.233047105  H,0,-0.2384667853,-3.0820362594,1.4777893702 |
|   Methoxy formate | C,0,-0.0448270076,0.179600751,1.0034830451  O,0,0.0803131812,1.0921475052,1.8176636431  O,0,-0.1405003194,0.1334628165,-0.2298536674  O,0,-0.0868843036,-1.1372419467,1.6769663822  C,0,-0.2279096904,-2.2226281529,0.8128786477  H,0,0.5955255285,-2.3048388388,0.0899711493  H,0,-1.1528016392,-2.1819887066,0.2209979787  H,0,-0.2459296289,-3.1322505214,1.4252912396 |
|   Methoxy carboxylic radical | C,0,-0.0493865413,0.0609096535,1.0539281353  O,0,0.0751521234,1.1072395027,1.7346480381  O,0,-0.1302670656,0.2253894696,-0.1794985279  O,0,-0.0863435671,-1.1109526117,1.6595328233  C,0,-0.2315828254,-2.2473802077,0.7870744012  H,0,0.6061241353,-2.3103135661,0.0944278911  H,0,-1.1632791809,-2.185982471,0.2270342538  H,0,-0.2434309589,-3.1126468801,1.4402514031 |
|   **TS6** | C,0,-0.067716935,0.309431377,1.1578034109  O,0,-0.6495805395,0.2573386699,2.2267772273  O,0,0.7609403237,0.8465149499,0.5097993208  O,0,-0.8571426113,-0.9199217579,0.4204238113  C,0,-0.2067861512,-2.1506747126,0.5444258405  H,0,-0.5385709558,-2.7771542791,-0.2868607252  H,0,-0.5310218796,-2.6495782072,1.4643772655  H,0,0.8812474487,-2.05084424,0.541514849 |
| CO2  Carbon dioxide | C,0,-0.005409772,-1.120327405,0.5410483176  O,0,-0.005409772,-1.120327405,-0.619142571  O,0,-0.005409772,-1.120327405,1.7012392063 |
| CH2O  Formaldehyde | C,0,0.,0.,1.0437786758  H,0,0.,0.9383659945,1.6407787059  H,0,0.,-0.9383659945,1.6407787059  O,0,0.,0.,-0.1520360876 |
| HO-CH2-OH  methanediol | C,0,0.1442743563,0.0212837632,1.1853772671  H,0,1.2263884332,0.1316936987,1.3093391887  H,0,-0.3531615606,0.9246765669,1.552755462  O,0,-0.1927489899,-1.1151721713,1.9330240413  H,0,-1.1516340489,-1.2035492909,1.8970110944  O,0,-0.2313542905,-0.077963279,-0.1611537093  H,0,0.2427262422,-0.8302130039,-0.5325510758 |
|   hydroxymethanolate | C,0,0.0989905444,0.0928593292,1.1327228242  H,0,1.2263568184,0.0298895293,1.2607267315  H,0,-0.1980021649,1.0786008357,1.6132411216  O,0,-0.389762881,-0.9198330987,2.1761704587  H,0,-0.9260416075,-1.4516602454,1.5746695472  O,0,-0.3697777095,-0.1488853502,-0.0411786833 |
|   Hydroxymethoxyl radical | C,0,0.0772844152,0.0383840498,1.1848257136  H,0,1.1940597073,0.0405454726,1.2305496699  H,0,-0.1687324631,1.0652404144,1.5513565447  O,0,-0.3892044205,-0.8812170579,2.1405008456  H,0,-0.961766081,-1.4928599264,1.6614778055  O,0,-0.309878158,-0.0891219525,-0.0523585793 |
|   Formate | C,0,0.0365064702,3.2820188216,0.5247376701  H,0,0.1526144498,3.1354536866,-0.6090216527  O,0,-0.149992875,4.4642440338,0.8784154008  O,0,0.1181773698,2.2320737319,1.1947503398 |
|  | C,0,0.0462796425,3.2696408049,0.4293694064  H,0,0.1574084721,3.1298675984,-0.6565552854  O,0,-0.1465383119,4.3834752479,0.9631058887  O,0,0.1001556121,2.3308066227,1.2529617483 |
| H2 | H,0,0.000001252,0.348605582,0.2519193239  H,0,0.000001252,0.348605582,-0.4919273139 |

# 6. Reference

[1] F. Neese, The ORCA program system. *WIREs Comput. Mol. Sci.* **2012**, *2*, 73-78.

[2] F. Neese, F. Wennmohs, U. Becker, C. Riplinger, The ORCA quantum chemistry program package. *The J. Chem. Phys.* **2020**, *152*, 224108.

[3] L. Simonelli, C. Marini, W. Olszewski, M. Ávila Pérez, N. Ramanan, G. Guilera, V. Cuartero, K. Klementiev, CLÆSS: The hard X-ray absorption beamline of the ALBA CELLS synchrotron. *Cogent Physics* **2016**, *3*, 1231987.

[4] B. Ravel, M. Newville, ATHENA, ARTEMIS, HEPHAESTUS: data analysis for X-ray absorption spectroscopy using IFEFFIT. *Synchrotron Radiat.* **2005**, *12*, 537-541.

[5] R. Sang, Z. Wei, Y. Hu, E. Alberico, D. Wei, X. Tian, P. Ryabchuk, A. Spannenberg, R. Razzaq, R. Jackstell, J. Massa, P. Sponholz, H. Jiao, H. Junge, M. Beller, Methyl formate as a hydrogen energy carrier. *Nat. Catal.* **2023**, *6,* 543-550.

[6] Y. Zhao, D. G. Truhlar, A new local density functional for main-group thermochemistry, transition metal bonding, thermochemical kinetics, and noncovalent interactions. *J. Chem. Phys.* **2006**, *125*, 194101.

[7] A. Schäfer, C. Huber, R. Ahlrichs, Fully optimized contracted Gaussian basis sets of triple zeta valence quality for atoms Li to Kr. *J. Chem. Phys.* **1994**, *100*, 5829-5835.

[8] A. V. Marenich, C. J. Cramer, D. G. Truhlar, Universal solvation model based on solute electron density and on a continuum model of the solvent defined by the bulk dielectric constant and atomic surface tensions. *The J. Phys. Chem. B* **2009**, *113*, 6378-6396.

[9] M. J. Frisch, G. W. Trucks, H. B. Schlegel, G. E. Scuseria, M. A. Robb, J. R. Cheeseman, G. Scalmani, V. Barone, G. A. Petersson, H. Nakatsuji, X. Li, M. Caricato, A. V. Marenich, J. Bloino, B. G. Janesko, R. Gomperts, B. Mennucci, H. P. Hratchian, J. V. Ortiz, A. F. Izmaylov, J. L. Sonnenberg, D. Williams-Young, F. Ding, F. Lipparini, F. Egidi, J. Goings, B. Peng, A. Petrone, T. Henderson, D. Ranasinghe, V. G. Zakrzewski, J. Gao, N. Rega, G. Zheng, W. Liang, M. Hada, M. Ehara, K. Toyota, R. Fukuda, J. Hasegawa, M. Ishida, T. Nakajima, Y. Honda, O. Kitao, H. Nakai, T. Vreven, K. Throssell, J. A. Montgomery, Jr., J. E. Peralta, F. Ogliaro, M. J. Bearpark, J. J. Heyd, E. N. Brothers, K. N. Kudin, V. N. Staroverov, T. A. Keith, R. Kobayashi, J. Normand, K. Raghavachari, A. P. Rendell, J. C. Burant, S. S. Iyengar, J. Tomasi, M. Cossi, J. M. Millam, M. Klene, C. Adamo, R. Cammi, J. W. Ochterski, R. L. Martin, K. Morokuma, O. Farkas, J. B. Foresman, D. J. Fox, Gaussian 16, revision C.01; Gaussian, Inc., Wallingford, CT, **2016**.

[10] G. Zhang, C. Zhang, H. Jiao, F. Chen, Photoredox/nickel cooperatively catalyzed radical allylic silylation of allyl acetates–Scope and mechanism. *J. Catal.* **2023**, *418*, 312-319.
